# Canonical strigolactones are not the major determinant of tillering but important rhizospheric signals in rice

**DOI:** 10.1126/sciadv.add1278

**Published:** 2022-11-02

**Authors:** Shinsaku Ito, Justine Braguy, Jian You Wang, Akiyoshi Yoda, Valentina Fiorilli, Ikuo Takahashi, Muhammad Jamil, Abrar Felemban, Sho Miyazaki, Teresa Mazzarella, Guan-Ting Erica Chen, Akihisa Shinozawa, Aparna Balakrishna, Lamis Berqdar, Chakravarty Rajan, Shawkat Ali, Imran Haider, Yasuyuki Sasaki, Shunsuke Yajima, Kohki Akiyama, Luisa Lanfranco, Matias D. Zurbriggen, Takahito Nomura, Tadao Asami, Salim Al-Babili

**Affiliations:** ^1^ Department of Bioscience, Faculty of Life Science, Tokyo University of Agriculture, 1-1-1 Sakuragaoka, Setagaya, Tokyo 156-8502, Japan.; ^2^ King Abdullah University of Science and Technology (KAUST), Biological and Environmental Sciences and Engineering Division, The BioActives Lab, Thuwal 23955-6900, Saudi Arabia.; ^3^ Center for Desert Agriculture, King Abdullah University of Science and Technology (KAUST), Thuwal, Saudi Arabia.; ^4^ Plant Science Program, Biological and Environmental Science and Engineering Division, King Abdullah University of Science and Technology (KAUST), Thuwal, Saudi Arabia.; ^5^ Institute of Synthetic Biology and CEPLAS, University of Düsseldorf, Universitätstrasse 1, Building 26.12.U1.25, Düsseldorf 40225, Germany.; ^6^ Department of Biological Production Science, United Graduate School of Agricultural Science, Tokyo University of Agriculture and Technology, 3-5-8 Saiwai-cho, Fuchu, Tokyo 183-8509, Japan.; ^7^ Department of Life Sciences and Systems Biology, University of Torino, Viale Mattioli 25, Torino 10125, Italy.; ^8^ Graduate School of Agricultural and Life Sciences, The University of Tokyo, 1-1-1 Yayoi, Bunkyo-ku, Tokyo 113-8657, Japan.; ^9^ Faculty of Science and Technology, Keio University, 3-14-1 Hiyoshi, Kohoku-ku, Yokohama 223-8522, Japan.; ^10^ Genome Research Center, Tokyo University of Agriculture, 1-1-1 Sakuragaoka, Setagaya, Tokyo 156-8502, Japan.; ^11^ Kentville Research and Development Centre, 32 Main Street, Kentville, NS B4N 1J5, Canada.; ^12^ Department of Applied Life Sciences, Graduate School of Life and Environmental Sciences, Osaka Prefecture University, Sakai, Osaka 599-8531, Japan.; ^13^ Center for Bioscience Research and Education, Utsunomiya University, 350 Minemachi, Utsunomiya, Tochigi 321-8505, Japan.

## Abstract

Strigolactones (SLs) are a plant hormone inhibiting shoot branching/tillering and a rhizospheric, chemical signal that triggers seed germination of the noxious root parasitic plant *Striga* and mediates symbiosis with beneficial arbuscular mycorrhizal fungi. Identifying specific roles of canonical and noncanonical SLs, the two SL subfamilies, is important for developing *Striga*-resistant cereals and for engineering plant architecture. Here, we report that rice mutants lacking canonical SLs do not show the shoot phenotypes known for SL-deficient plants, exhibiting only a delay in establishing arbuscular mycorrhizal symbiosis, but release exudates with a significantly decreased *Striga* seed–germinating activity. Blocking the biosynthesis of canonical SLs by TIS108, a specific enzyme inhibitor, significantly lowered *Striga* infestation without affecting rice growth. These results indicate that canonical SLs are not the determinant of shoot architecture and pave the way for increasing crop resistance by gene editing or chemical treatment.

## Teaser

Canonical strigolactones are rhizospheric signals and do not significantly contribute to tillering inhibition in rice.

## INTRODUCTION

Strigolactones (SLs) are carotenoid-derived hormones characterized by an enol-ether bridge connecting a lactone ring (D-ring; fig. S1) (*1*) in *R* configuration to a structurally variable second moiety that consists of a tricyclic lactone ring (ABC-ring) in canonical SLs, while noncanonical SLs have variable structures based on a β-ionone ring (A-ring) (fig. S1) (*2*). SLs are a major determinant of plant architecture and are involved in many other biological processes. Among other phenotypes, mutants affected in SL biosynthesis are characterized by increased branching/tillering, shorter shoots (dwarf), and decreased primary root length (*2*–*4*).

In addition, when exposed to nutrients deficiency, in particular phosphate, plant roots release SLs to attract arbuscular mycorrhiza fungi (AMF) for establishing the plant-AMF symbiosis, the most common type of plant symbiosis that significantly increases the uptake of nutrients and water from the soil (*5*–*7*). However, canonical SLs, the first found type of these signaling molecules, were identified as the host-derived signal that stimulates seed germination in root parasitic weeds, such as *Orobanche* and *Striga* spp. (*8*). These weeds are obligate parasites that developed the ability to sense SLs as a seed germination signal enabling the coordination of their life cycle with the presence of an available host in the close vicinity (*9*). Infestation by root parasitic plants, such as *Striga hermonthica*, is a severe problem for agriculture and a major threat for global food security, in particular in Africa where it causes more than U.S. $7 billion annual losses in cereal production (*10*, *11*).

The availability of high-branching mutants of monocot and dicot plant species (*12*–*16*) paved the way for finding the hormonal function of SLs and enabled later the elucidation of their biosynthesis (fig. S2). SL biosynthesis starts with the reversible isomerization of all-*trans-* into 9-*cis*-β-carotene, catalyzed by DWARF27 (*17*–*18*). This step is followed by cleavage and rearrangement reactions mediated by the CAROTENOID CLEAVAGE DIOXYGENASE 7 and 8 (CCD7 and CCD8), which yield carlactone (CL), the core intermediate of SL biosynthesis (*18*, *19*). The discovery of CL unraveled the presence of the noncanonical SLs that were unknown before. Different modifications of CL, which are catalyzed by cytochrome P450 monooxygenases (CYP), in particular MORE AXILLARY GROWTH1 (MAX1) from the CYP711A clade, and other enzymes, give rise to the structural diversity of the more than 30 natural canonical and noncanonical SLs (*16*, *20*–*22*).

**Fig. 1.**
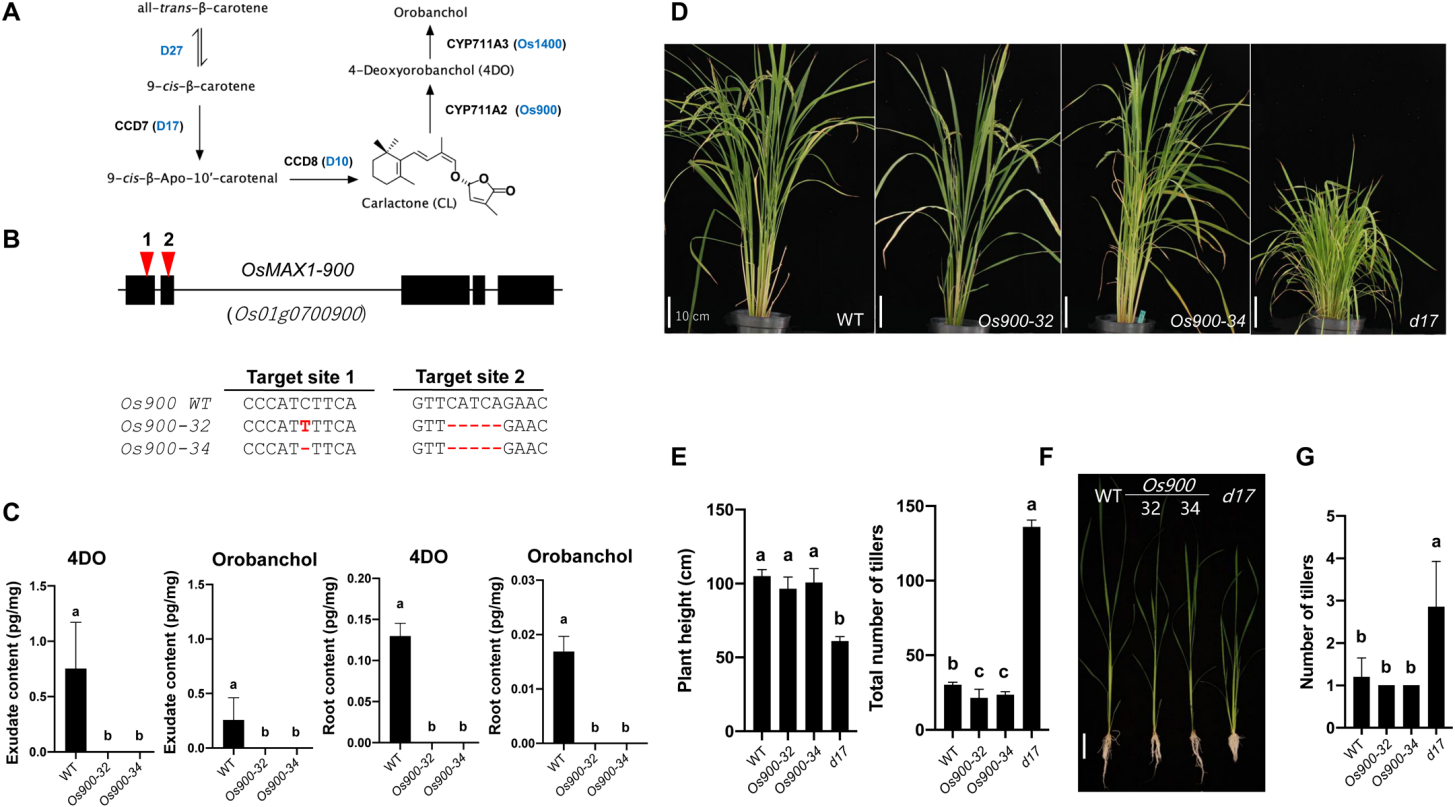
Generation of the *Os900*-KO lines by CRISPR-Cas9 system. (**A**) Scheme of the biosynthesis of the rice canonical SLs (the detailed SL biosynthesis pathway depicted in figs. S2 and S28). (**B**) The structure of the *Os900* gene and the sequences of the two CRISPR-Cas9 target sites indicated by red arrowheads. Details of the CRISPR-mediated mutations of the two KO lines, *Os900-*32 and *Os900-*34, are reported. (**C**) Analysis of canonical SLs, 4DO and orobanchol, in root exudates of *Os900*-KO lines grown under constant low-Pi conditions. The data are presented as means ± SD from five samples. Means not sharing a letter in common differ significantly at *P*_0.05_. (**D** and **E**) Shoot phenotypes of WT, *Os900-*KO lines, and *d17* mutant grown in soil and (**F** and **G**) hydroponic culture under +Pi conditions. Scale bars, 10 cm. The data are presented as means ± SD for the number of biological replicates [5 ≤ *n* ≤ 7 for WT, *Os900-32* and *Os900-34*, *n* = 3 for *d17* in (C) and (D); 4 ≤ *n* ≤ 8 for (F) and (G)].

Rice contains five *MAX1* homologs: *Os01g0700900* (*OsMAX1-900*), *Os01g0701400* (*OsMAX1-1400*), *Os01g0701500* (*OsMAX1-1500*), *Os02g0221900* (*OsMAX1-1900*), and *Os06g0565100* (*OsMAX1-5100*) (*23*, *24*), with a truncated *OsMAX1-1500* in the Nipponbare cv. (*24*). In vitro studies and transient expression in *Nicotiana benthamiana* showed that all functional Nipponbare OsMAX1 enzymes (OsMAX1-900, OsMAX1-1400, OsMAX-1900, and OsMAX1-5100) can convert CL into carlactonoic acid (CLA) that is transformed into the canonical SLs 4-deoxyorobanchol (4DO) and then orobanchol by sequential action of OsMAX1-900 and OsMAX1-1400 (Fig. 1A) (*25*, *26*). In this work, we investigated the biological function of canonical SLs in rice.

## RESULTS AND DISCUSSION

For this purpose, we generated two biallelic homozygote *OsMAX1-900* knockout (KO) lines (*Os900*-KO: *Os900-32* and *Os900-34*) disrupted in the biosynthesis of 4DO and orobanchol, the only rice canonical SLs (*1*), through introducing CRISPR-Cas9–induced deletion, point mutation, and frameshift mutations (Fig. 1B). We first quantified 4DO and orobanchol in roots and root exudates of hydroponically grown and phosphate-starved mutants by liquid chromatography–tandem mass spectrometry (LC-MS/MS) (Fig. 1C and fig. S3, A and B). 4DO and orobanchol were undetectable in both lines, confirming in planta the role of OsMAX1-900 as the rice 4DO synthase (*25*, *26*) and that 4DO is the exclusive precursor of orobanchol in rice. Besides the absence of 4DO and orobanchol, exudates of the mutant lines showed around 96% decrease in the content of a noncanonical SL tentatively identified as methyl 4-oxo-carlactonoate (4-oxo-MeCLA) (fig. S3C). This noncanonical SL, previously described as methoxy-5-deoxystrigol isomer (*27*), was also not detectable in the *Os900*-KO roots (fig. S3C). On the basis of the ion peak characteristic of the D-ring at 97.028, we also identified a novel SL “CL + 30” with a molecular formula C_19_H_24_O_5_ [mass/charge ratio (*m*/*z*) 333.16989 as positive ion [M + H]^+^, calculated for *m*/*z* 333.16965], which was present at high levels in the *Os900* mutants (Fig. 1D and fig. S3C). Feeding *Os900*-34 seedlings with [^13^C]-labeled CL confirmed that CL + 30 is a downstream product of CL (fig. S4); however, the enzyme responsible for the production of this metabolite remains elusive. To check the possibility that rice plants may compensate for the absence of functional MAX1-900 by increasing the expression of other MAX1 homologs and such increase is the reason for the accumulation of CL + 30, we determined the level of their transcripts in mutant roots. However, we did not detect significant changes in level of these transcripts, compared to wild type (WT; fig. S5). Hence, we do not have any hint for the involvement of MAX1 enzymes in the formation of CL + 30. The higher accumulation of CL + 30 in *Os900*-KO lines (fig. S3B) indicated that it might be a substrate of OsMAX1-900. We confirmed this assumption by expressing OsMAX1-900 in yeast cells and feeding them with a CL + 30–containing fraction. After incubation and LC-MS/MS analysis, we detected a reduction in CL + 30 content and its conversion into a novel metabolite eluting at 6.1 min (*m*/*z* 347 in positive ion mode and 345 in negative ion mode), corresponding to CL + 30 + 14 Da (CL + 30 + 14) (fig. S6). As OsMAX1-900 catalyzes the carboxylation of CL, we expected the arising metabolite to contain a carboxyl group. Therefore, we methylated the novel OsMAX1-900 product by diazomethane, which gave rise to a derivative with an *m*/*z* 361 in positive ion mode and fragment pattern and retention time (9.1 min) that are characteristic for the tentative 4-oxo-MeCLA (fig. S7). Given that OsMAX1-900 catalyzes the oxidation at the C19 position, we assumed that CL + 30 corresponds to 4-oxo-19-hydroxy-CL (fig. S8).

Next, we phenotyped the growth and development of the *Os900*-KO lines, in comparison with WT and the high-tillering SL-deficient *d17* mutant (*28*). In soil and under normal conditions, shoots of mature *Os900*-KO plants did not differ significantly from WT, in contrast to *d17* that showed the characteristic dwarfism and extreme high tillering (Fig. 1, D and E). *Os900*-KO lines had even less tillers, compared to WT (an average of 30 tillers for WT versus 21.4 and 23.6 tillers for *Os900-32* and *Os900-34*, respectively) (Fig. 1E and fig. S9A). *Os900*-KO mutants, grown in rhizotrons under normal conditions, showed a higher number of crown roots and root area, compared to WT (fig. S9, B and C). When hydroponically grown under different conditions (+Pi, −Pi, and low Pi), we did not detect common significant differences in shoot and root phenotype between the two mutants and the WT, apart from shorter shoots, lighter shoot, and a change in root biomass under both +Pi and −Pi conditions (Fig. 1, F and G, and fig. S10). In conclusion, we did not detect pronounced morphological alterations, which are characteristic for SL-deficient mutants, in the *Os900*-KO mutants in all three experiments, indicating that (i) canonical SLs are not a major regulator of rice architecture and (ii) the *Os900*-KO mutant lines still maintain a largely normal SL hormone homeostasis. To check the first assumption, we fed hydroponically grown *d17* seedlings with different concentrations of 4DO (0, 1, 10, 100, and 1000 nM) under normal conditions, using 1000 nM *rac-*GR24 (SL analog) as a positive control (fig. S11A) and determined the effect of the treatment on their phenotype (*29*). We observed a decrease in tillering to one to two tillers only at higher concentrations (100 and 1000 nM; fig. S11B). These concentrations appear much higher than endogenous SL levels (usually at picomolar level under nutrient deficiency conditions) (*30*). However, it is difficult to estimate the endogenous concentration of 4DO after this treatment, in the absence of knowledge about the absorbance and transport efficiency of exogenously applied 4DO. Hence, the biological functions of 4DO within rice plants remain elusive. For the second hypothesis, we treated *Os900*-KO mutants with 2.5 μM zaxinone, a growth-promoting apocarotenoid that requires intact SL biosynthesis and perception for its activity (*30*). As the root growth promoting activity of zaxinone was not observed in SL biosynthesis and perception mutants (*30*), we checked whether this apocartenoid can promote the growth of *Os900*-KO mutants that lack canonical SLs. Zaxinone application significantly enhanced root and shoot length of *Os900*-KO lines (fig. S12), suggesting that SL hormone biosynthesis and signaling are working properly in the absence of 4DO and orobanchol and that zaxinone does not depend on canonical SLs in its growth-promoting activity. In conclusion, these data suggest that canonical SLs are not a major determinant of the tiller number in rice, which is in line with a recently published study on the role of orobanchol in tomato (*22*). Moreover, our results indicate that noncanonical SLs are likely the SL hormone regulating shoot architecture. The absence of canonical SLs in the shoot base, where tillers emerge, of WT and the *Os900* mutants that do not show shoot architecture–related phenotypes, and the accumulation of CL + 30 as the only SL in shoot base (root-shoot junction) of these mutants (figs. S13 to S15) support this assumption. Analysis of the transcript levels of the three intact *MAX1* homologs in roots, shoot base, and leaves of *Os900* mutants did not provide any indication that the activity of the missing *OsMAX1-900* is compensated by the induction of any of them (figs. S5 and S16). Hence, understanding the weak low-tillering phenotype of the *Os900* mutants and the biosynthesis and conversion of CL + 30 requires further investigations. The finding that canonical SLs do not significantly contribute to the regulation of tillering in planta does not exclude that they can bind to SL receptors and trigger SL signal transduction when applied exogenously at sufficient concentrations. *rac*-GR24, which can be considered as an analog of canonical SLs, is generally used to study SL hormonal functions and the mechanisms of SL perception and signaling (*2*, *4*, *31*). The usage of GR24 for such studies is a result of being the first and most available SL analog. However, the discovery of CL and noncanonical SLs has led to the development of corresponding analogs, such as methyl phenlactonoate 3 (MP3) and MP7, which show higher activity in inhibiting rice tillering, compared to *rac*-GR24 (*29*).

**Fig. 2.**
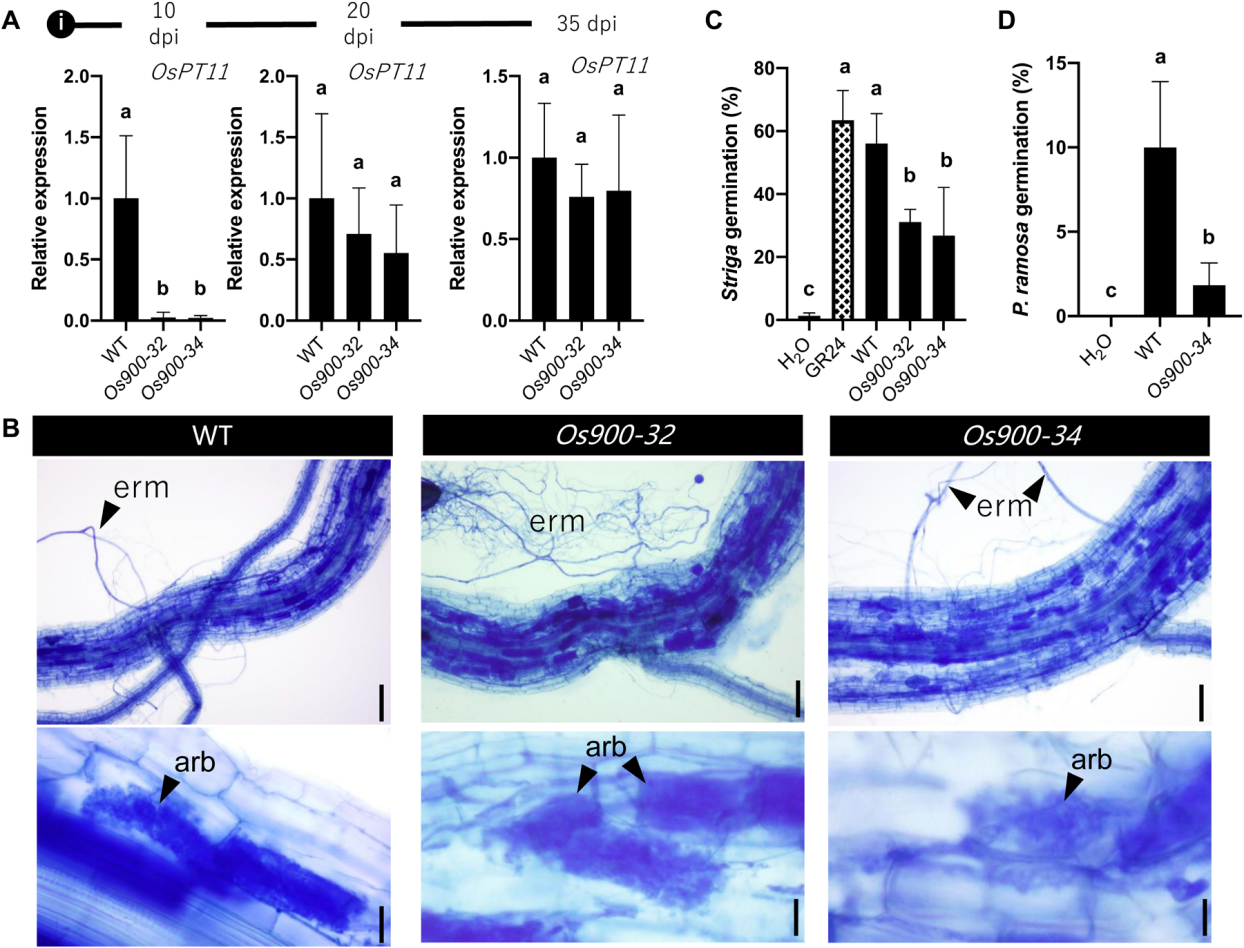
Evaluation of rhizospheric interactions. Effect of *Os900*-KO lines on the arbuscule formation (**A** and **B**) and the germination of root parasitic weeds [(**C**) *Striga* and (**D**) *Phelipanche*]. The values are represented as the means ± SD for the number of biological replicates [(A) and (B), *n* = 4; (C) 2 < *n* < 4; and (D) *n* = 3]. Scale bars, 10 μm. The statistical significance is determined by one-way analysis of variance (ANOVA) and Tukey’s multiple comparison test. Arbuscule formation of *R. irregularis* was quantified by measuring the expression of marker gene (*OsPT11*) (A). (B) Arbuscule formation at 35 dpi. Arb, arbuscule- containing cells; Erm, extraradical mycelium.

Next, we investigated the role of canonical SLs as rhizospheric signals. First, we estimated the colonization of *Os900*-KO roots by the AMF *Rhizophagus irregularis* after 10, 20, and 35 days post-inoculation (dpi). For this purpose, we used the transcript level of *OsPT11*, an AMF-specific phosphate transporter gene (*32*). At 10 dpi, there was a delay in colonization of *Os900*-KO roots compared to WT roots, whereas, at 20 and 35 dpi, the colonization of *Os900* mutants was comparable to the WT (Fig. 2A). No other phenotypic differences were observed in the colonization itself (Fig. 2B and fig. S17). Moreover, application of *Os900*-KO root exudates to *Gigaspora margarita* spores triggered a comparable germination rate as *rac*-GR24 (fig. S18). This result suggests that noncanonical SLs are present at relatively higher level in exudates of *Os900*-KO plants, compared to WT, enabling them to sustain the capability to induce AMF spore germination in the absence of canonical SLs. Noncanonical SLs, such as CLA, were shown to have substantial hyphal branching activity in AMF (*33*). Recent studies on SL biosynthesis and function in *Marchantia paleacea* unraveled the formation of a noncanonical SL, bryosymbiol, and indicated that the evolutionary original function of SLs is related to the communication with AMF rather than plant architecture regulation (*34*). We also tested the activity of *Os900*-KO lines root exudates in inducing germination of *S. hermonthica* and *Phelipanche ramosa* seeds and observed more than 50% decrease in the germination of both parasitic species, compared to WT exudates (Fig. 2, C and D, and fig. S19). This indicates that 4DO and orobanchol are important cues for parasitic seed germination, especially 4DO that was shown to be a stronger germination signal than orobanchol (*35*).

Together, we can conclude that the two rice canonical SLs, 4DO and orobanchol, are rhizospheric signals that play a role in AM symbiosis. Decreasing their level or even completely knocking out their biosynthesis can significantly reduce the damage caused by *Striga* and other root parasitic plants, without causing severe plant architectural changes or having large negative impact on mycorrhization. However, modulation of SL content by genetic engineering or gene editing requires years of development, while application of specific inhibitors of their biosynthesis may lead much faster to rice plants lacking 4DO and orobanchol. Therefore, we set out to identify chemical(s) that inhibit canonical SL biosynthesis in rice.

**Fig. 3.**
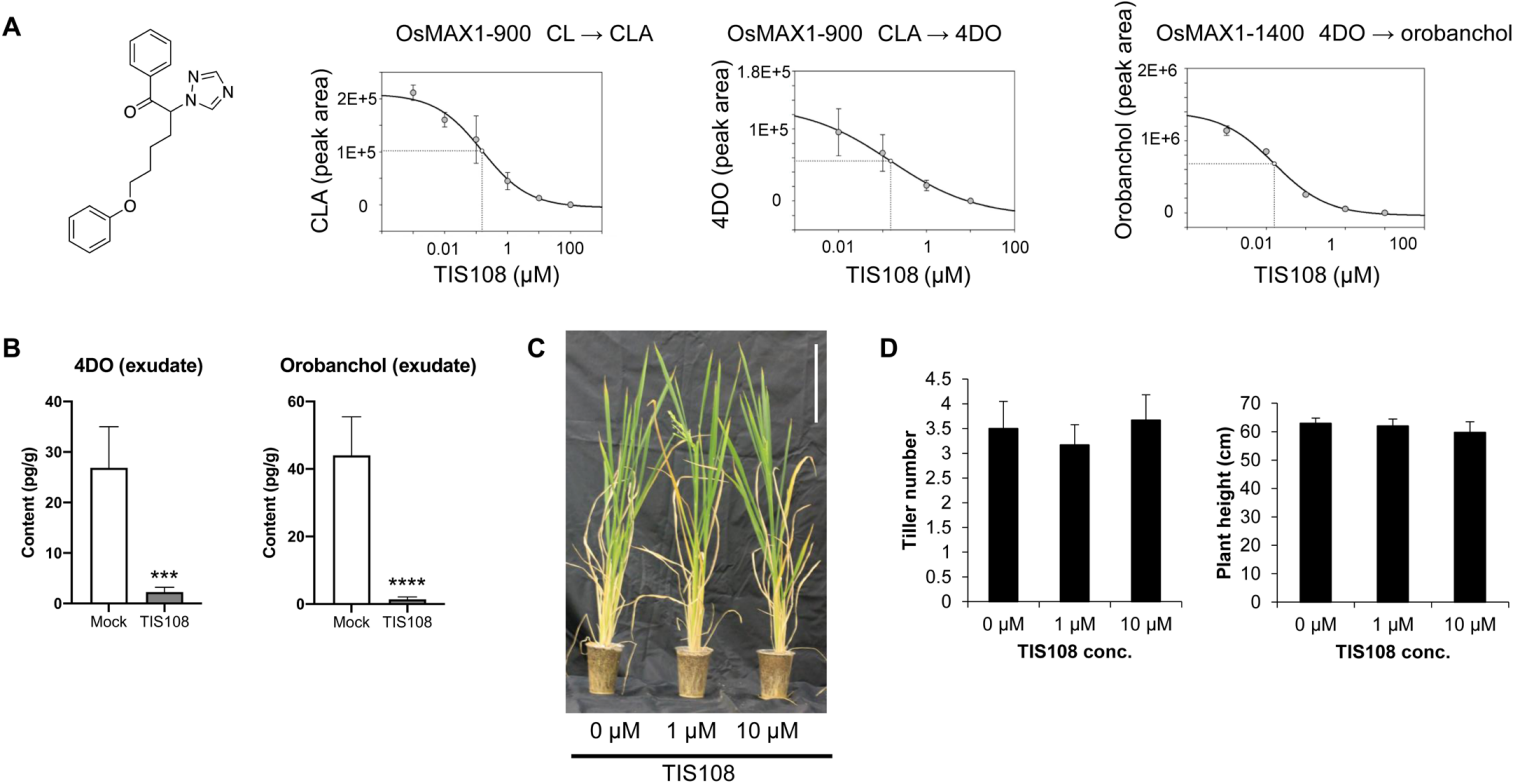
TIS108 is an OsMAX1 inhibitor. (**A**) Structure of TIS108 and inhibition of the activity of rice MAX1s by TIS108. Different substrates (CL, CLA, and 4DO) and concentrations of TIS108 were incubated with MAX1-containing yeast microsomes. Assay extracts and authentic standard controls were analyzed by liquid chromatography–tandem mass spectrometry. (**B**) TIS effect on canonical SLs, 4DO and orobanchol, in root exudates of WT grown under constant low-Pi conditions. The data are presented as means ± SD of five biological replicates. Asterisk indicates significant difference without (mock) and with 10 μM TIS108 treatment (TIS108) (***P < 0.001 and ****P ≤ 0.0001, Student’s *t* test). (**C**) Three-month-old rice plants treated with TIS108. Scale bar, 10 cm. (**D**) Tiller number and plant height of plants from (C).

TIS108 is an inhibitor of SL biosynthesis, which contains a 1*H*-1,2,4-triazole moiety (Fig. 3A) that can bind to the heme iron of P450s, such as MAX1 enzymes, and potentially impede their function (*36*). TIS108 inhibited the conversion of CL to CLA to 4DO by OsMAX1-900 [half maximal inhibitory concentration (IC_50_) = 0.15 μM, for both conversions], and of 4DO to orobanchol by OsMAX1-1400 (IC_50_ = 0.02 μM) (Fig. 3A), when added to assays with microsomes prepared from yeast cells overexpressing the corresponding MAX1 enzyme. We could not determine whether TIS108 also affects the activity of OsMAX1-5100 and OsMAX1-1900, as we did not detect the reported conversion of CL to CLA, neither with native nor with codon-optimized OsMAX1-5100 and OsMAX1-1900 (fig. S20).

**Fig. 4.**
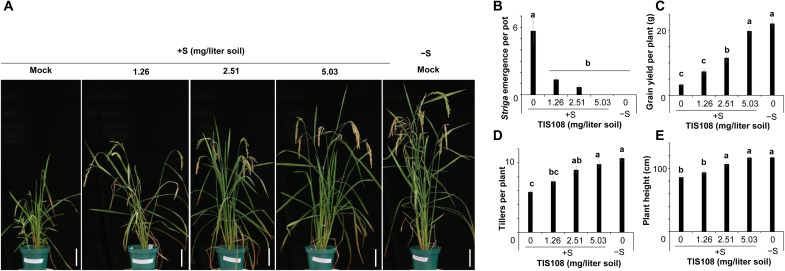
Application of TIS108 mitigates *Striga* infestation. (**A** to **E**) *Striga* emergence test in rice grown in the presence (+S) or absence (−S) of *Striga* seeds for 8 weeks. The soil was treated with 0, 10, 20, or 40 μM TIS108 once a week up to 3 weeks. Total amounts of TIS108 were 1.26 (10 μM TIS108), 2.51 (20 μM TIS108), and 5.03 mg/liter (40 μM TIS108) soil. (B) Number of emerged *Striga* plants after 8 weeks. Grain yield (C), number of tillers (D), and plant height (E) were recorded at final harvesting. Scale bars, 20 cm. The data are presented as means ± SE from six samples. Different letters indicate statistically significant differences at *P*_0.05_.

To confirm the effect of TIS108 on the biosynthesis of canonical SLs in planta and to check its impact on plant growth and architecture, we applied the inhibitor to hydroponically grown rice seedlings under phosphate starvation. TIS108 treatment caused a significant decrease of 4DO, orobanchol, and 4-oxo-MeCLA level and an accumulation of CL + 30 (Fig. 3B and fig. S21). Seedlings of the rice *d14*-1 SL-perception mutant, which contains higher amounts of SLs due to the absence of a negative feedback regulation, showed similar responses to TIS108 treatment, i.e., a decrease of canonical SLs in roots and root exudates and an enhancement in CL + 30 level (fig. S22). The application of TIS108 to 2-week-old rice WT seedlings grown in hydroponics (fig. S23) or soil (Fig. 3, C and D) did not cause phenotypic alterations, compared to the mock. We also investigated the effect of TIS108 on rice transcriptome, using RNA sequencing (data file S1). None of the identified 174 up-regulated and 107 down-regulated differentially expressed genes in TIS108-treated rice (tables S1 and S2) was related to tillering or SL biosynthesis. Although the effect of TIS-108 on meristematic area might be plausible, this result is in line with the absence of significant morphological changes upon TIS108 treatment (table S3). Furthermore, we investigated the impact of TIS108 on AM symbiosis. Application of this inhibitor at a 10 μM concentration to plants grown in sand containing *R. irregularis* unraveled a colonization pattern similar to that observed with the *Os900*-KO mutants, reported through the expression level of *OsPT11* transcript. TIS108 caused a delay in mycorrhization at 10 dpi, which was recovered at 20 dpi. However, by the end of the experiment, TIS108-treated plants showed a tendency toward reduction of *OsPT11* transcript level, compared to WT (fig. S24). Next, we investigated whether TIS108 can be used for reducing *Striga* infestation. For this purpose, we exposed rice grown in *Striga*-infested soil to TIS108 at concentrations of 0, 0.0782, 0.235, and 0.782 mg/liter (total amounts) over a 7-week time period. Results obtained showed a reduction of *Striga* emergence in a dose-dependent manner (Fig. 4, A to E, and figs. S25 and S26). We did not observe this decrease when we added the SL analog MP1 (*29*) to the TIS108 treatment, suggesting that the lower *Striga* emergence detected with TIS108 alone is a result of lower level of germination stimulants in the root exudates. Lower infestation protected the rice plants from *Striga*-induced growth inhibition (Fig. 4A), leading to number of tillers and spikes, plant height, grain yield, and grain number similar to those of WT rice grown in *Striga*-free soil and without TIS108 treatment (Fig. 4, B to E, and figs. S24 and S25). We also tested the effect of TIS108 on Indica rice and sorghum, major crops in *Striga*-infested regions in Africa. Here again, we observed lower *Striga* germination–inducing activity of the exudates isolated from TIS108-treated plant rice (fig. S27). Overall, the application of TIS108 mimics the effect of knocking out *MAX1-900* in the *Os900* mutants (fig. S28), with respect to the level of canonical SLs and biological activity of root exudates, suggesting that rice canonical SLs are rhizospheric signals rather than the tillering-inhibitory hormone.

Together, we used genetic and chemical strategies to manipulate rice SL compositions, which allowed us to distinguish the biological functions of canonical and noncanonical SLs in rice. Our findings unraveled the possibility of reducing *Striga* infestation by gene editing or chemical treatment without significantly affecting host’s morphology and growth or its ability to establish AM symbiosis. Direct application of TIS108 and using gene editing represent promising strategies for alleviating the threat posed by *Striga* and other root parasitic plants to global food security.

## MATERIALS AND METHODS

### Plant and fungi materials

Rice (*Oryza sativa*, cv. Nipponbare) mutant lines were generated in previous studies: *d17* (*dl-2*) (*28*) and *d10* (cv. Shiokari) (*36*). Their respective WT backgrounds were used in the experiments.

*R. irregularis* DAOM 197198 was obtained from Agronutrition, in Labège, France. *S. hermonthica* seeds were collected from sorghum fields during the 2012 rainy season in Sudan and were provided by A. Gabbar Babiker. *P. ramosa* seeds were provided by P. Semier, Université de Nantes, France.

### Generation of *Os900*-KO plants

Rice (*O. sativa* L. ssp. japonica cv. Nipponbare) *OsMAX1-900* (*Os01g0700900*/JX235697) gene was targeted by CRISPR-Cas9 guided by two guide RNAs [gRNAs; single gRNA1 (sgRNA1), 5′-ggaagtacggccccatcttc-3′ and sgRNA2, 5′-aattctcctgttcatcagaa-3′], designed using the CRISPR-PLANT database (*28*). The construction of the tRNA-gRNA-Cas9 cassette was accomplished through Golden Gate assembly into pRGEB32 binary vector containing hygromycin resistance gene (*29*). Induced Nipponbare calli, from mature seeds, were transformed with *Agrobacterium tumefaciens* EHA105 culture containing the plasmid of interest, selected and regenerated in the presence of hygromycin. After successive regeneration of shoots and roots in a Percival growth chamber (CLF Plant Climatics GmbH, model CU 36L5), the plantlets were transferred to soil and grown in a greenhouse at 28°C day/22°C night (*30*).

Plant transgenicity was tested by polymerase chain reaction (PCR) amplification of the region surrounding the two sgRNA insertion sites in the pRGEB32 vector with pRGEB32-specific primers pRGEB32-F (5′-ccacgtgatgtgaagaagtaagataaactg-3′) and pRGEB32-R (5′-gataggtttaagggtgatccaaattgagac-3′). The CRISPR-mediated mutations were identified by amplifying the DNA region that contains the sgRNA target sites with the genome-specific primers Os900 sg2-sg3 F (5′-gccatactggaaagtgcgg-3′) and Os900 sg2-sg3 R (3′-tagcttcaggtaaaattgcgcg-5′) and by sequencing the resulting 455-bp-long PCR fragment (fig. S29).

The potential off-target effects of the sgRNA3 and sgRNA4 have been investigated using the online tool CRISPR-P 2.0 (http://cbi.hzau.edu.cn/cgi-bin/CRISPR2/CRISPR) (fig. S30 and table S5).

### Hydroponic culture of rice seedlings

Seeds from T_3_ homozygous plants were first surface-sterilized in 2% sodium hypochlorite (v/v) with 0.01% Tween 20 for 20 min under gentle agitation before being rinsed generously with sterile water and germinated overnight in the dark (30°C). The pregerminated seeds were then transferred to round petri dishes containing two sheets of sterile Whatman filter paper and 5 ml of half-strength Murashige and Skoog (MS) (PhytoTechnology Laboratories, catalog no. M519) solution (pH 5.7) and incubated for another 2 days in the dark at 30°C. Last, 15 ml of modified Hoagland nutrient solution adjusted to pH 5.8 [0.4 mM K_2_HPO_3_·3H_2_O, 5.6 mM NH_4_NO_3_, 0.8 mM MgSO_4_·7H_2_O, 0.8 mM K_2_SO_4_, 0.18 mM FeSO_4_·7H_2_O, 0.18 mM Na_2_EDTA·H_2_O, 1.6 mM CaCl_2_, 0.8 mM KNO_3_, and micronutrients (0.023 mM H_3_BO_3_, 4.5 μM MnCl_2_·4H_2_O, 0.3 μM CuSO_4_·5H_2_O, 1.5 μM ZnCl_2_, and 0.1 μM Na_2_MoO_4_·2H_2_O)] was added, and seedlings were incubated in a Percival for 5 days (day/night temperature of 28°/22°C and a 12-hour photoperiod and 200 μmol photons m^−2^ s^−1^).

The setup of the hydroponic culture consisted of 50-ml black tubes with a perforated cap containing in its center a 1.5-ml bottomless Eppendorf tube, into which the 1-week-old seedlings were transferred. The nutrient solution provided with (+Pi) or without (−Pi) 0.4 mM K_2_HPO_3_·3H_2_O. –Pi conditions were achieved by feeding the seedlings with +Pi for 2 weeks, followed by 1 week of −Pi treatment before further analysis (seedling phenotyping). The solutions were changed every 3 days.

For low-Pi conditions, the same procedure was performed but with replacing the half-strength MS solution by 5 ml of modified Hoagland nutrient solution adjusted to pH 5.8 and containing 0.004 mM K_2_HPO_3_·3H_2_O (low Pi), right after the sterilization step. Plants were kept for 3 weeks in low-Pi solution.

### Phenotyping in pots and rhizotron

For phenotyping of *Os900* mutants, seedlings were transferred into pots filled with soil containing half-strength modified Hoagland nutrient solution. The nutrient solution consisted of 5.6 mM NH_4_NO_3_, 0.8 mM MgSO_4_.7H_2_O, 0.8 mM K_2_SO_4_, 0.18 mM FeSO_4_.7H_2_O, 0.18 mM Na_2_EDTA.2H_2_O, 1.6 mM CaCl_2_.2H_2_O, 0.8 mM KNO_3_, 0.023 mM H_3_BO_3_, 0.0045 mM MnCl_2_.4H_2_O, 0.0003 mM CuSO_4_.5H_2_O, 0.0015 mM ZnCl_2_, 0.0001 mM Na_2_MoO_4_.2H_2_O, and with or without 0.4 mM K_2_HPO_4_.2H_2_O, resulting in the +Pi and −Pi medium, respectively. The pH of the solution was adjusted to 5.8, and the solution was applied every third day. On day 56, phenotypic data were recorded. The plants were grown in a greenhouse from February to April 2020, in Thuwal (Saudi Arabia).

For observing the root phenotypes of the *Os900* mutants in the rhizotron system (48 cm by 24 cm by 5 cm), 3-day-old seedlings were grown in soil with Hoagland nutrient solution containing 0.4 mM K_2_HPO_4_.2H_2_O (+Pi) for 2 weeks. The solution was changed every other day with fresh nutrient solution. Root length, angle, and surface area were analyzed with the ImageJ software.

### Qualitative and quantitative analysis of SLs in root exudates, root tissues, leaves, and shoot base

Analysis of SLs in rice root exudates and root tissues was performed following the protocol described by Wang *et al.* (*30*, *37*). Briefly, collected 50-ml root exudates of two seedlings grown together in one tube and spiked with 0.672 ng of D_6_-5DS were brought on a C_18_-Fast Reversed-Phase SPE column (500 mg/3 ml; GracePure) preconditioned with 3 ml of methanol and 3 ml of water. After washing with 3 ml of water, SLs were eluted with 5 ml of acetone. The SL fraction was concentrated to SL aqueous solution (∼1 ml), followed by 1 ml of ethyl acetate extraction. SL-enriched organic phase (750 μl) was dried under vacuum. For analysis of root tissues, around 25 mg of lyophilized and grinded root tissues spiked with 0.672 ng of D_6_-5DS was extracted twice with 2 ml of ethyl acetate in an ultrasound bath (Branson 3510 ultrasonic bath) for 15 min, followed by centrifugation for 8 min at 3800 rpm at 4°C. The two supernatants were combined and dried under vacuum. The residue was dissolved in 50 μl of ethyl acetate and 2 ml of hexane, followed by a Silica Cartridges SPE column (500 mg/3 ml; HyperSep) purification. After washing with 3 ml of hexane, SLs were eluted in 3 ml of ethyl acetate and evaporated to dryness under vacuum. The same procedure was used for shoot base (shoot-root junction) extraction, except for the tissue powder preparation: 12 fresh shoot bases were pulled together and manually grinded while preserved in liquid nitrogen. The entire sample was used during the extraction. The final extract was redissolved in 100 μl of acetonitrile:water [25:75 (v/v)] and filtered through a 0.22-μm filter for LC-MS/MS analysis.

SLs were identified by using the UHPLC-Orbitrap ID-X Tribrid Mass Spectrometer (Thermo Scientific Altis) with a heated-electrospray ionization source (H-ESI). Chromatographic separation was achieved on the Hypersil GOLD C_18_ Selectivity HPLC Column (150 mm by 4.6 mm; 3 μm; Thermo Fisher Scientific) with mobile phases consisting of water (A) and acetonitrile (B), both containing 0.1% formic acid, and the following linear gradient (flow rate, 0.5 ml/min): 0 to 15 min, 25 to 100% B, followed by washing with 100% B and equilibration with 25% B for 3 min. The injection volume was 10 μl, and the column temperature was maintained at 30°C for each run. The MS conditions were as follows: positive mode, ion source of H-ESI; spray voltage of 3500 V; sheath gas flow rate of 60 arbitrary units; auxiliary gas flow rate of 15 arbitrary units; sweep gas flow rate of 2 arbitrary units; ion transfer tube temperature of 350°C; vaporizer temperature of 400°C; S-lens radio-frequency level of 60; resolution of 120,000 for MS; stepped stepped collision energy (HCD) of 10, 20, 30, 40, 50%; and resolution of 30,000 for MS/MS. The mass accuracy [accurate mass ± 5 parts per million (ppm) mass tolerance] of identified compounds and their MS spectra (accurate mass ± 5 ppm mass tolerance) were acquired using Xcalibur software version 4.1.

SLs were quantified by LC-MS/MS using a high-performance liquid chromatography (HPLC)–triple quadrupole/linear ion trap instrument (QTRAP5500; AB Sciex) and UHPLC-Triple-Stage Quadrupole Mass Spectrometer (Thermo Scientific Altis). Chromatographic separation was achieved on a ZORBAX Eclipse plus C_18_ column (150 mm by 2.1 mm; 3.5 μm; Agilent) with mobile phases consisting of water:acetonitrile [95:5 (v/v); A] and acetonitrile (B), both containing 0.1% formic acid, and the following linear gradient (flow rate, 0.5 ml/min): 0 to 15 min, 25 to 100% B, followed by washing with 100% B and equilibration with 25% B for 3 min. The injection volume was 10 μl, and the column temperature was maintained at 35°C for each run. The MS parameters of QTRAP5500 were as follows: positive ion mode, ion source of turbo spray, ion spray voltage of 5500 V, curtain gas of 40 psi, collision gas of medium, gas 1 of 60 psi, gas 2 of 50 psi, turbo gas temperature of 400°C, declustering potential of 60 V, entrance potential of 10 V, collision energy of 16 eV, and collision cell exit potential of 10 V. The MS parameters of Thermo Scientific Altis were as follows: positive ion mode, ion source of H-ESI, ion spray voltage of 5000 V, sheath gas of 40 arbitrary units, auxiliary gas of 15 arbitrary units, sweep gas of 2 arbitrary units, ion transfer tube gas temperature of 350°C, vaporizer temperature of 350°C, collision energy of 17 eV, collision-induced dissociation (CID) gas of 2 mtorr, and full width at half-maximum 0.2 Da of Q1/Q3 mass. The characteristic multiple reaction monitoring transitions (precursor ion → product ion) were 331.15 → 216.0, 331.15 → 234.1, and 331.15 → 97.02 for 4DO; 347.14 → 329.14, 347.14 → 233.12, 347.14 → 205.12, and 347.14 → 97.02 for orobanchol; 361.16 → 247.12, 361.16 → 177.05, 361.16 → 208.07, and 361.16 → 97.02 for 4-oxo-MeCLA; 333.17 → 219.2, 333.17 → 173.2, 333.17 → 201.2, and 333.17 → 97.02 for putative 4-oxo-hydroxyl-CL (CL + 30); and 337.19 → 222.15, 337.19 → 240.16, and 337.19 → 97.02 for D_6_-5-deoxystrigol.

### Feeding with^13^C-labeled CL

^13^C-CL was prepared by following the protocol described by Bruno *et al.* (*19*). Briefly, the *OsCCD8* complementary DNA (cDNA), controlled by an arabinose-inducible promoter, was expressed as thioredoxin-fusion in BL21 Rosetta *Escherichia coli* cells. A single colony of the transformed *E. coli* was cultured overnight, from which 0.5 ml was inoculated into 50 ml of media and grown at 28°C. When the optical density at 600 nm = 0.5, we induced the protein production with 0.2% (w/v) arabinose and incubated under agitation for 4 hours at 28°C. The harvested cells (centrifugation) were resuspended in lysis buffer [sodium phosphate buffer pH 8 containing 1% Triton X-100 and 10 mM dithiothreitol (DTT), and lysozyme (1 mg/ml)] and incubated on ice for 30 min. The crude lysate was then sonicated and centrifuged at 12,000 rpm and 4°C for 10 min. The protein was collected (supernatant) to be used for the in vitro incubation with ^13^C-labeled 9-cis-β-apo-10-carotenal, from Buchem B. V. (Apeldoorn, the Netherlands). The substrate (^13^C-labeled 9-cis-β-apo-10-carotenal) was quantified spectrophotometrically, dried, and resuspended in ethanolic detergent mixture 0.4% (v/v) Triton X-100. The mixture was then dried using a vacuum centrifuge to produce a carotenoid-containing gel, which was resuspended in incubation buffer [2 mM tris 2-carboxyethylphosphine, 0.4 mM FeSO_4_, and catalase (2 mg/ml; Sigma-Aldrich, Deisenhofen, Germany) in 200 mM Hepes/NaOH, pH 8]. OsCCD8 crude cell lysate, i.e., 50 μl of the soluble fraction of overexpressing cells, was added to the assay, and the whole mix was incubated 4 hours under shaking at 140 rpm at 28°C in the dark. The reaction was stopped by adding two volumes of acetone, and the lipophilic compounds were separated by partition extraction with petroleum ether:diethyl ether 1:4 (v/v), dried, and resuspended in methanol for HPLC analysis.

^13^C-CL was preparatively purified using a YMC-Pack C30–reversed-phase column [250 mm by 4.6 mm inner diameter (i.d.), 5 μm] in Agilent 1260 HPLC. The following separation systems were used. The column was developed at a flow rate of 1 ml/min with a gradient from 100% B to 80% B [MeOH:water:tert-butylmethyl ether (30:10:1, v/v/v)] within 15 min, then to 100% A [methanol:tert-butylmethyl ether (1:1, v/v)] within 0.5 min, and last to 100% A and a flow rate of 2 ml/min within 0.5 min, maintaining the final conditions for another 14 min. The collected fractions were dried under nitrogen gas, dissolved in dichloromethane, and kept at −80°C. Around 20 ng of ^13^C-CL was fed to 2-week-old *Os900* rice seedlings for 6 hours, and then 500 ml of root exudates was collected for LC-MS/MS analysis.

### Gene expression analysis

For transcript analysis, total RNA was extracted from rice roots, leaves, and shoot base using the Direct-zol RNA Miniprep Plus kit (Zymo Research, #R2071), according to the manufacturer’s instructions. cDNA was synthetized from 2 μg of total RNA using the iScript cDNA Synthesis kit (Bio-Rad Laboratories Inc., 2000 Alfred Nobel Drive, Hercules, CA, USA) according to the instructions in the user manual. Quantitative reverse transcription PCR (qRT-PCR) was performed using SYBR Green Master Mix (Applied Biosystems), which was diluted before use. Each PCR was carried out in a total volume of 10 μl containing 4 μl of diluted cDNA (the resulting cDNA was diluted 1:10 in H_2_O), 5 μl of 2× diluted SYBR Green Reaction Mix, and 0.5 μl of each primer (2 μM working stock). All reactions were performed on a 384-well plate in a CFX384 Touch Real-Time PCR Detection System (Bio-Rad) as follows: 95°C for 90 s, 40 cycles of 95°C for 15 s, and 60°C for 30 s. All reactions were performed on at least three biological and three technical replicates, including a water control to exclude potential unspecific amplification. The 2−^ΔΔ^*C*_T_ method was used to calculate the relative gene expression levels (*38*), and rice ubiquitin (*OsUBQ*) gene was used as the internal control to normalize target gene expression (see fig. S28 for primer sequences).

For *OsPT11* gene expression analysis (AMF marker), total RNA was extracted from rice roots using the Plant RNeasy Kit (Qiagen), according to the manufacturer’s instructions. Samples were treated with TURBO DNase (Ambion) according to the manufacturer’s instructions. The RNA samples were routinely checked for DNA contamination by means of PCR analysis, using primers for *OsRubQ1* (*32*). For single-strand cDNA synthesis, about 1000 ng of total RNA was denatured at 65°C for 5 min and then reverse-transcribed at 25°C for 10 min, 42°C for 50 min, and 70°C for 15 min. The reaction was carried out in a final volume of 20 μl containing 10 μM random primers, 0.5 mM deoxynucleotides (dNTPs) 4 μl 5× buffer, 2 μl 0.1 M DTT, and 1-μl SuperScript II (Invitrogen). qRT-PCR was performed using a Rotor-Gene Q 5plex HRM Platform (Qiagen). Each PCR was carried out in a total volume of 15 μl containing 2 μl of diluted cDNA (about 10 ng), 7.5 μl of 2× SYBR Green Reaction Mix, and 2.75 μl of each primer (3 μM). The following PCR program was used: 95°C for 90 s, 40 cycles of 95°C for 15 s, and 60°C for 30 s. A melting curve (80 steps with a heating rate of 0.5°C per 10 s and a continuous fluorescence measurement) was recorded at the end of each run to exclude the generation of nonspecific PCR products. All reactions were performed on at least three biological and three technical replicates. Baseline range and take-off values were automatically calculated using Rotor-Gene Q 5plex software. Transcript level of *OsPT11* (*32*) were normalized using *OsRubQ1* housekeeping gene (*32*). Only take-off values leading to a *C*_t_ mean with a SD below of 0.5 were considered.

### Exogenous applications of 4DO and zaxinone

For investigating the effect of zaxinone (customized synthesis from Buchem B.V.; Apeldoorn, the Netherlands) on different genotypes, 1-week-old seedlings were grown hydroponically in half-strength Hoagland nutrient solution containing 0.4 mM K_2_HPO_4_·2H_2_O (+Pi), 2.5 μM zaxinone (dissolved in 0.1% acetone), 1 μM *rac*-GR24 (purchased from StrigoLab, Turin, Italy), or the corresponding volume of the solvent (mock; acetone) for 2 weeks. The solution was changed twice per week, adding the chemical at each renewal.

### Plant material and growth conditions for *R. irregularis* root colonization

Seeds of WT plants and *Osmax1*-independent lines (*Os900-32* and *Os900-34*) were germinated in pots containing sand and incubated for 10 days in a growth chamber under a 14-hour light (23°C)/10-hour dark (21°C). Plants used for mycorrhization were inoculated with ~1000 sterile spores of *R. irregularis* DAOM 197198 (Agronutrition, Labège, France). A set of WT mycorrhizal plants were treated with TIS108 (10 μM), once per week, by applying the compound once a week directly in the nutrient solution. Nonmycorrhizal and mycorrhizal plants were grown in sterile quartz sand and watered with a modified Long-Ashton (LA) solution containing 3.2 μM Na_2_HPO_4_·12H_2_O (*39*). Mycorrhizal level was monitored during a time course experiment from 10 to 35 dpi. In the last time point (35 dpi), WT and *Os900* mycorrhizal roots were stained with 0.1% cotton blue in lactic acid and the estimation of mycorrhizal parameters was performed by the Trouvelot method (*40*). Four parameters were considered: *F*%, percentage of segments showing internal colonization (frequency of mycorrhization); *M*%, average percentage of colonization of root segments (intensity of mycorrhization); *a*%, percentage of arbuscules within infected areas; and *A*%.

For the phenotype evaluation of nonmycorrhizal and mycorrhizal plants, we considered the following parameters: crown root number, root and shoot length, leaves number, and root and shoot fresh weight. Data are means ± SE (*n* ≤ 10). To analyze the fungal intraradical morphology, root apparatus was stained in cotton blue [0.1% (w/v)] in lactic acid, cut in pieces 1 cm long, and observed under an optical microscope.

### Investigation of TIS108 effect on WT plants and *R. irregularis* root colonization

Seeds of WT plants and *Os900* mutants *cv* Nipponbare were germinated in pots containing sand and incubated for 10 days in a growth chamber under a 14-hour light (23°C)/10-hour dark (21°C). Plants used for mycorrhization were inoculated with ~1000 sterile spores of *R. irregularis* DAOM 197198 (Agronutrition, Labège, France). A set of WT mycorrhizal plants were treated with TIS108 (10 μM) by applying the compound once a week directly in the nutrient solution. Plants were grown in sterile quartz sand and were watered with a modified LA solution containing 3.2 μM Na_2_HPO_4_·12 H_2_O. Mycorrhizal level was monitored during a time course experiment at 10, 20, and 35 dpi.

### Gene expression analysis of mycorrhizal plants

Total RNA was extracted from rice roots using the Plant RNeasy Kit (Qiagen), according to the manufacturer’s instructions. Samples were treated with TURBO DNase (Ambion) according to the manufacturer’s instructions. The RNA samples were routinely checked for DNA contamination by means of PCR analysis, using primers for *OsRubQ1* (*32*). For single-strand cDNA synthesis, about 1000 ng of total RNA was denatured at 65°C for 5 min and then reverse-transcribed at 25°C for 10 min, 42°C for 50 min, and 70°C for 15 min. The reaction was carried out in a final volume of 20 μl containing 10 μM random primers, 0.5 mM dNTPs, 4 μl of 5× buffer, 2 μl of 0.1 M DTT, and 1 μl of SuperScript II (Invitrogen). qRT-PCR was performed using a Rotor-Gene Q 5plex HRM Platform (Qiagen). Each PCR was carried out in a total volume of 15 μl containing 2 μl of diluted cDNA (about 10 ng), 7.5 μl of 2× SYBR Green Reaction Mix, and 2.75 μl of each primer (3 μM). The following PCR program was used: 95°C for 90 s, 40 cycles of 95°C for 15 s, and 60°C for 30 s. A melting curve (80 steps with a heating rate of 0.5°C per 10 s and a continuous fluorescence measurement) was recorded at the end of each run to exclude the generation of nonspecific PCR products. All reactions were performed on at least three biological and three technical replicates. Baseline range and take-off values were automatically calculated using Rotor-Gene Q 5plex software. Transcript level of OsPT11 (*32*) were normalized using *OsRubQ1* housekeeping gene (*32*). Only take-off values leading to a *C*_t_ mean with an SD below 0.5 were considered. Statistical tests were carried out through one-way analysis of variance (ANOVA) and Tukey’s post hoc test, using a probability level of *P* < 0.05. All statistical elaborations were performed using PAST statistical [version 2.16; (*41*)].

### Spore germination assay

Spores were sterilized in a solution of streptomycin sulfate [0.03% (w/v)] and chloramine T [3% (w/v)] and germinated in 100 μl of *max1-32* and *max1-34* root exudates at 10^−9^ M (CL + 30 content was determined by D_6_-5DS), GR24 10^−9^ M, or a solution of water/acetone (0.08%). For each treatment, 96 sterilized spores were placed individually in the wells of a multiwell plate and treated with freshly prepared solutions at the beginning of the experiment. Spores were germinated in the dark at 30°C, and the germination rate was evaluated after 3 days.

### *S. hermonthica* and *P. ramosa* germination bioassays

*S. hermonthica* and *P. ramosa* seeds were first sterilized and preconditioned as described before (*42*). The preconditioned seeds were spread on a 9-mm-diameter Whatman filter paper disc. Each disc contains from 40 up to 100 seeds. Then, five discs were moved to a plastic petri dish, where each disc was used as a technical replicate. Eluted in acetone (the same procedure was followed for root exudates’ extracts to be used for bioassay experiments, omitting the addition of labeled SL as the internal standard), the root exudates were added to water (300 μl of sample in 300 μl of H_2_O) to allow acetone evaporation under vacuum centrifugation while minimizing sample degradation and was applied on each disc containing pregerminated seeds. Corresponding volumes of *rac*-GR24 (purchased from StrigoLab, Turin, Italy) and sterile MilliQ water were included as positive and negative control, respectively, knowing that seeds would germinate only in the presence of SL-like compounds. The petri dishes were sealed with parafilm, enfolded with aluminum foil and incubated for 24 hours at 30°C for *Striga* and 3 days at 28°C for *Phelipanche*. The germinated and nongerminated *Striga* seeds–containing discs were photographed using a Leica LED3000 R binocular microscope, adjusted to 50% medium light, mounted with a charge-coupled device camera (Leica Microsystems). The germination percentage of the acquired images was assessed by the seed counter software SeedQuant (*43*).

### Heterologous expression of SL biosynthetic genes in yeast

Heterologous expressions of *Os900* and *Os1400* in yeast (*Saccharomyces cerevisiae*) was carried out as described previously (*26*). Yeast microsomes (approximately 100 μg of proteins/100 μl) were incubated with 0.001 to 1000 μM TIS108 [1 μM substrate *rac*-CL and 500 μM NADPH (reduced form of nicotinamide adenine dinucleotide phosphate)] at 28°C for 1 min. The reaction was stopped with the addition of 1 ml of ethyl acetate. The ethyl acetate phase was dried with anhydrous sodium sulfate and then subjected to LC-MS/MS analysis as described previously (*26*, *44*). The products CLA, 4DO, and orobanchol were quantified from peak areas in the transitions of *m*/*z* 331.1 to 113.0 in negative mode, *m*/*z* 331.1 to 97.0 in positive mode, and *m*/*z* 347.0 to 97.0 in positive mode, respectively. The IC_50_ values were obtained using triplicate samples and calculated with SigmaPlot software (Systat Software Inc., CA, USA).

### Phenotyping TIS108-treated rice

Rice (*O. sativa* L. “IAC-165”) cultivation and chemical treatment was performed for 2 weeks as previously described (*35*). For prolonged cultivation, 2-week-old rice seedlings were planted in vermiculite and grown under the same conditions, TIS108 (100 mM) was diluted 1:10,000 from an acetone-dissolved stock solution in a hydroponic culture solution (*3*), and 100 ml was applied to the pots once a week for 10 weeks. Acetone was used as the control treatment.

### RNA sequencing

Rice was grown under the same conditions as describe for the SL analysis. Fourteen-day-old seedlings were transferred to a brown vial with or without 10 μM TIS108 for 1 day. Roots were harvested and total RNA was extracted using an RNA purification reagent (Invitrogen, USA). The quality of total RNA was evaluated using an Agilent 2100 Bioanalyzer (Agilent, USA). Each library was prepared with NEB Next Ultra II RNA Prep Kit for Illumina according to the manufacturer’s protocol (New England Biolabs, USA). The quality of each library was assessed using an Agilent 2100 Bioanalyzer and then sequenced using an Illumina NextSeq Sequencer (single-end sequencing, 75 bp). The datasets from this sequence have been deposited in the DNA Data Bank of Japan database (accession number: DRA009250). Total reads were mapped to the rice transcripts using Bowtie (*45*). Differential gene expression was examined using DESeq2 (*46*) and established by fold change (log_2_ ratio) and false discovery rate (FDR) (log_2_ ratio ≧ 1 and FDR ≦ 0.05).

### *S. hermonthica* pot test

Seeds of *S. hermonthica* (3 mg) were sown in plastic pots (70 mm i.d. and 84 mm in height) containing 150 ml of soil [Bonsol No.2 (Sumitomo Chemical, Osaka, Japan) and river sand = 1:1 (v/v)] and 60 ml of distilled water. Seeds in the pots were conditioned in the dark at 30°C for 7 days. On the eighth day, one 5-day-old rice seedling (*O. sativa* L. IAC-165) was then planted in each *S. hermonthica* conditioned pot and grown in growth chamber (NK System, Tokyo, Japan) at 30°C under light-emitting diode light (500 μmol m^−2^ s^−1^) with long-day conditions (16-hour light/8-hour dark). Each pot was treated with 50 ml of 0.1, 0.3, or 1 μM TIS108 and MP1 (*29*, *47*, *48*) once a week for 7 weeks. The total amount of TIS108 applied was 0.0782, 0.235, and 0.782 mg/liter soil. After 8 weeks, the number of *S. hermonthica* plants that emerged from each pot was counted and photographically recorded.

For *S. hermonthica* pot test, seeds of *S. hermonthica* (20 mg) were thoroughly mixed in 1.5 liters of sand and soil mixture (1:1) and added to a 3-liter perforated plastic pot containing 0.5-liter clean soil in the bottom. The pots were kept in greenhouse-controlled conditions at 30°C in moist conditions for 10 days to precondition the *Striga* seeds. On the 11th day, 5-day-old rice seedlings (*O. sativa* L. IAC-165) were planted in the middle of each pot. After 3 days, each pot was treated with 250 ml of TIS108 at 10, 20, or 40 μM as an irrigation application. The compound was applied once a week for 3 weeks. The total amount of TIS108 applied was 0.84, 1.68, and 3.35 mg/liter soil. The untreated pots with (+S) and without (−S) *Striga* were included as control treatments. The number of emerged *Striga* plants was recorded from each pot up to 8 weeks after initial *Striga* emergence. Rice growth, yield, and yield components were observed at the time of final harvesting.

### Statistical analysis

Data are represented as means and their variations as SD. The statistical significance was determined by one-way ANOVA and Tukey’s multiple comparison test, using a probability level of *P* < 0.05. All statistical elaborations were performed using GraphPad Prism 8 for Mac OS, version 8.3.0.
