# Supplementary material for: Canonical strigolactones are not the major determinant of tillering but important rhizospheric signals in rice
Source: Sci Adv. 2022 Nov 2;8(44):eadd1278. doi: 10.1126/sciadv.add1278 (PMC9629705; doi:10.1126/sciadv.add1278)
Supplement: Supplementary file 1 — Figs. S1 to S30 Tables S1 to S5 [file sciadv.add1278_sm.pdf]

Science Advances
AAAS

Supplementary Materials for

Canonical strigolactones are not the major determinant of tillering but important rhizospheric signals in rice

Shinsaku Ito *et al.*

Corresponding author: Salim Al-Babili, salim.babili@kaust.edu.sa; Tadao Asami, asami@g.ecc.u-tokyo.ac.jp; Takahito Nomura, tnomura@cc.utsunomiya-u.ac.jp

Sci. Adv. **8**, eadd1278 (2022)
DOI: 10.1126/sciadv.add1278

The PDF file includes:

Figs. S1 to S30
Tables S1 to S5
Legend for data S1

Other Supplementary Material for this manuscript includes the following:

Data S1

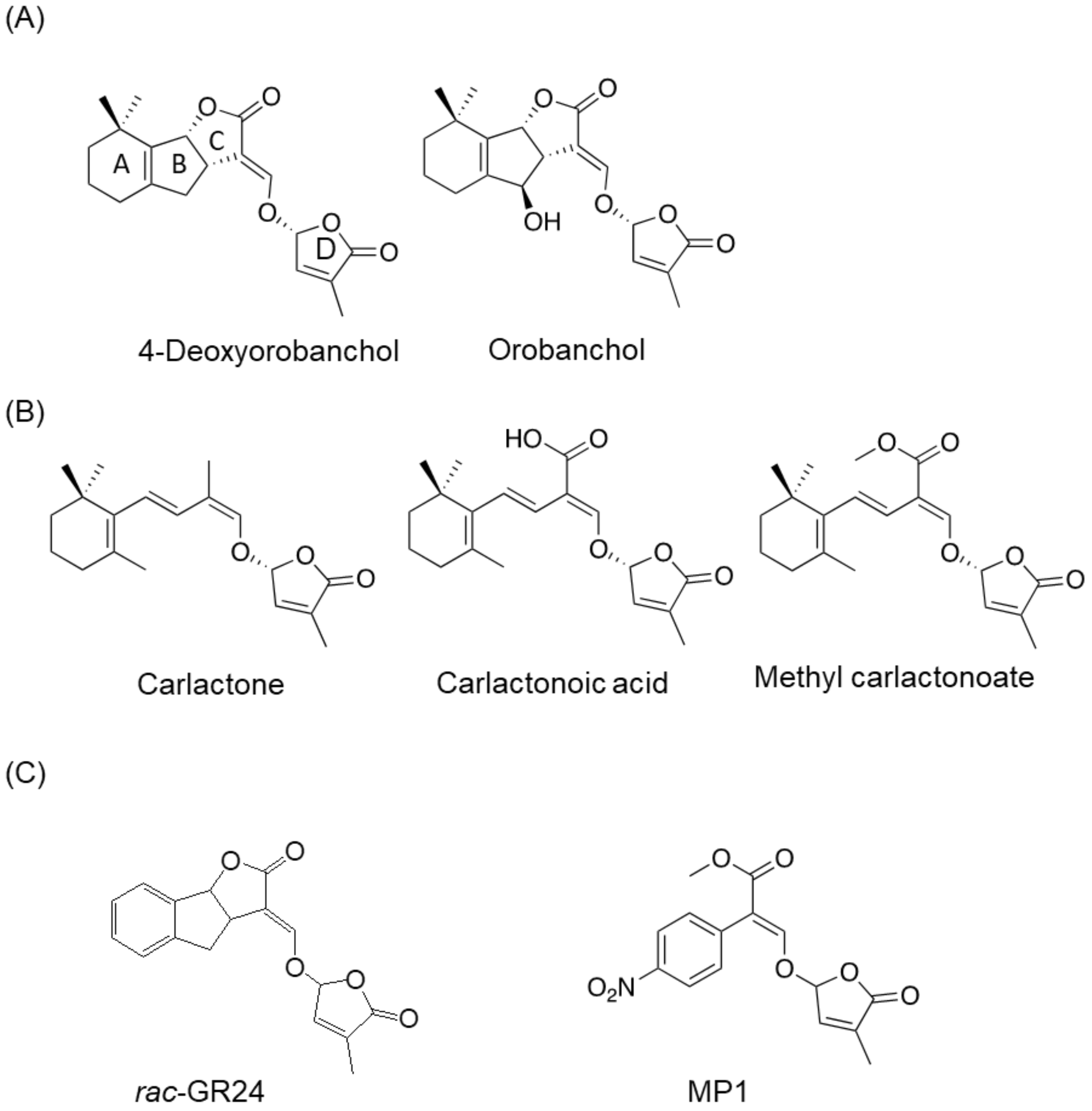

Fig. S1.
Structures of canonical SLs (**A**), non-canonical SLs (**B**), and SL analogs used in this study (**C**).

Abbreviations: MP1, Methyl Phenlactonoate 1

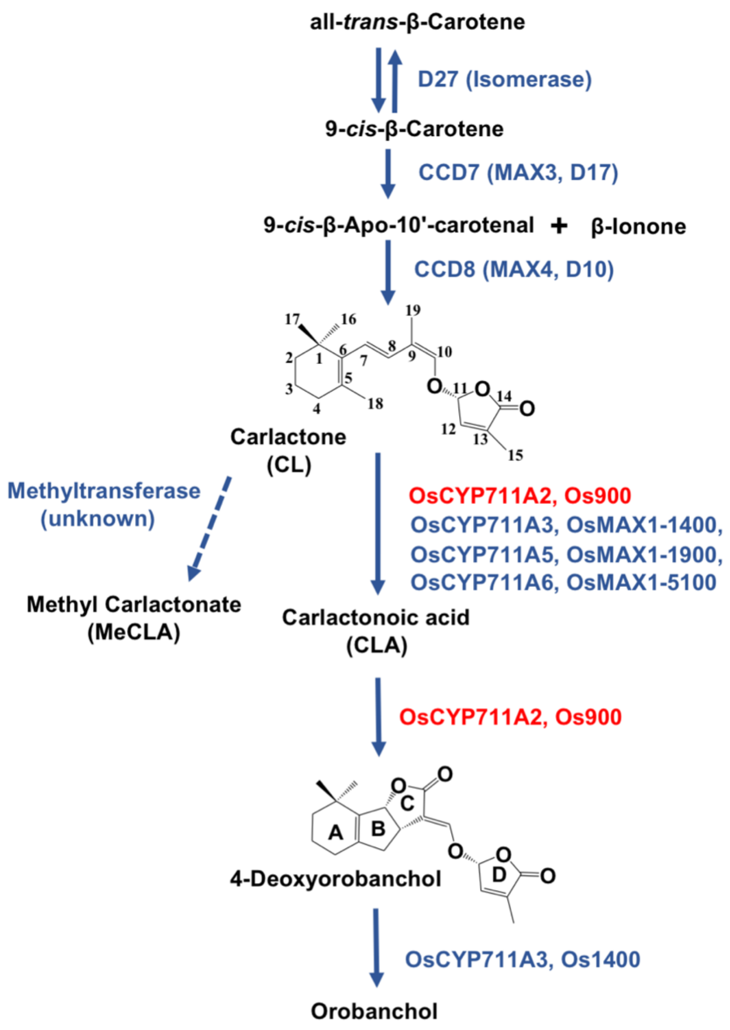

Fig. S2.
SL biosynthetic pathway in rice.

Abbreviations: D27, Dwarf27; CCD, Carotenoid Cleavage Dioxygenase; MAX1, More Axillary Growth 1; CYP, Cytochrome P450

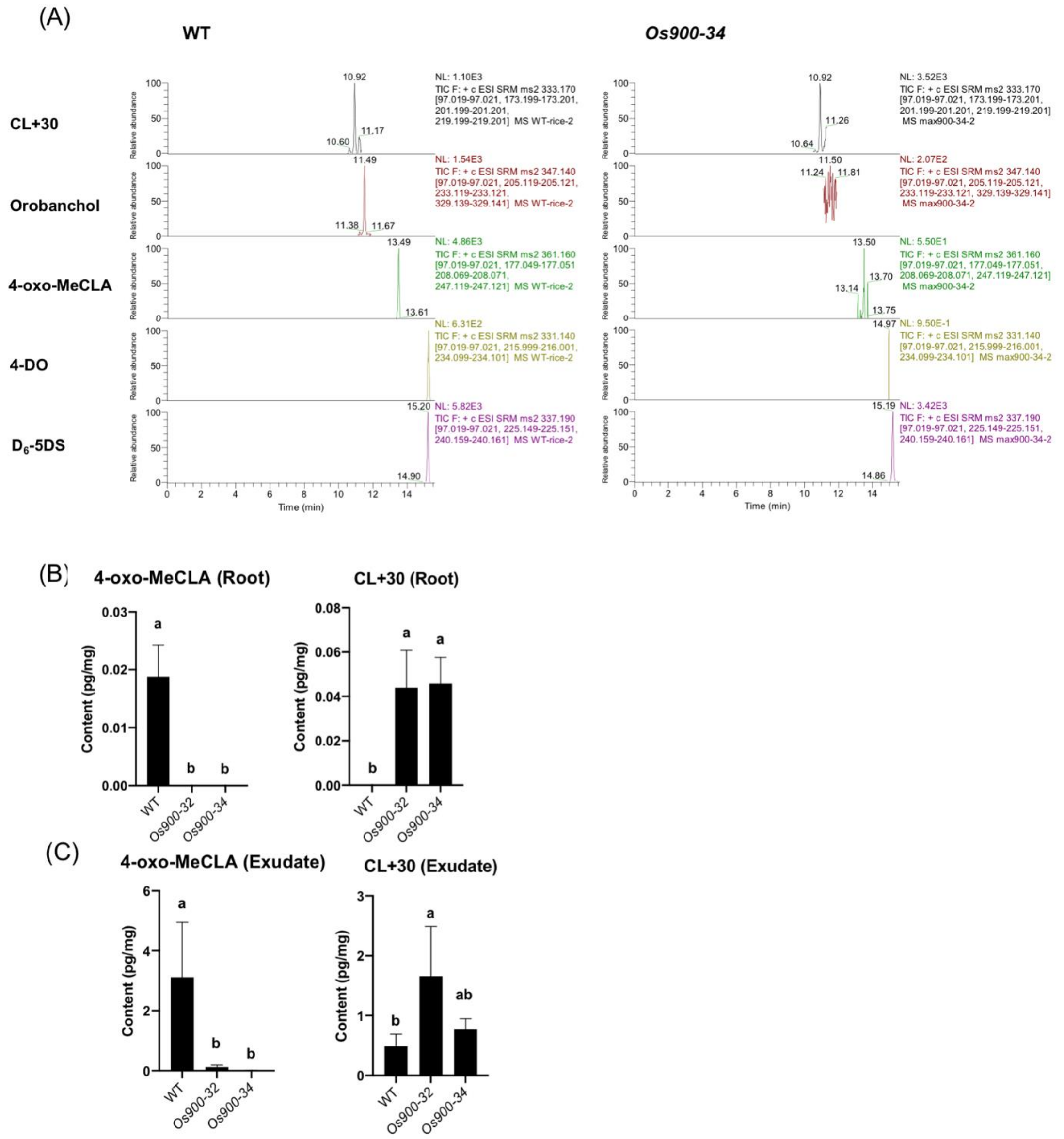

Fig. S3.

Identification and quantification different rice SLs. Multiple reaction monitoring (MRM) chromatograms used to identify and quantify SLs in root exudates of WT and *Os900-34* plants grown under low Pi conditions (**A**). Quantification of putative non-canonical SLs, i.e. a tentative methyl 4-oxo-carlactonoate (4-oxo-MeCLA; structure proposed by Yoneyama et al., 2018) and CL+30, in root tissues (**B**) and root exudates (**C**) of *Os900*-KO and WT plants grown under constant low Pi conditions. The data are presented as means ± SD of five biological samples. Means lacking a common letter differ significantly at $P_{0.05}$. Abbreviations: CL, carlactone; 4-oxo-MeCLA, methyl 4-oxo-carlactonoate; 4-DO, 4-deoxyorobanchol; 5-DS, 5-deoxystrigol; WT, wild-type.

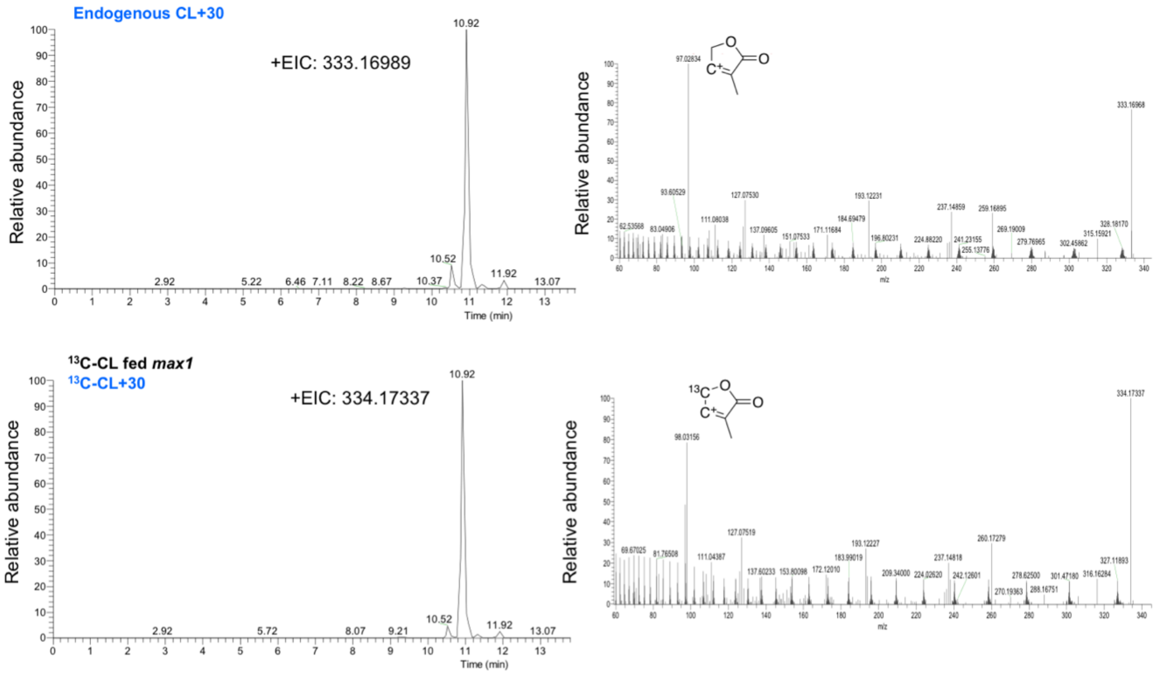

Fig. S4.

Conversion of ^{13}C-labbeled CL to CL+30. Identification of endogenous CL+30 (tentatively 4-oxo-hydroxyl-CL) (Retention time: 10.89). Product ion spectra derived from the precursor ion (*m/z* 333.16968 $[M+H]^+$ in positive mode) with characterized D-ring at *m/z* 333.16968>97.02834. [^{13}C]-CL+30 was characterized using an ion pair at *m/z* 334.17337>98.03156. The proposed structures of fragments are inserted.

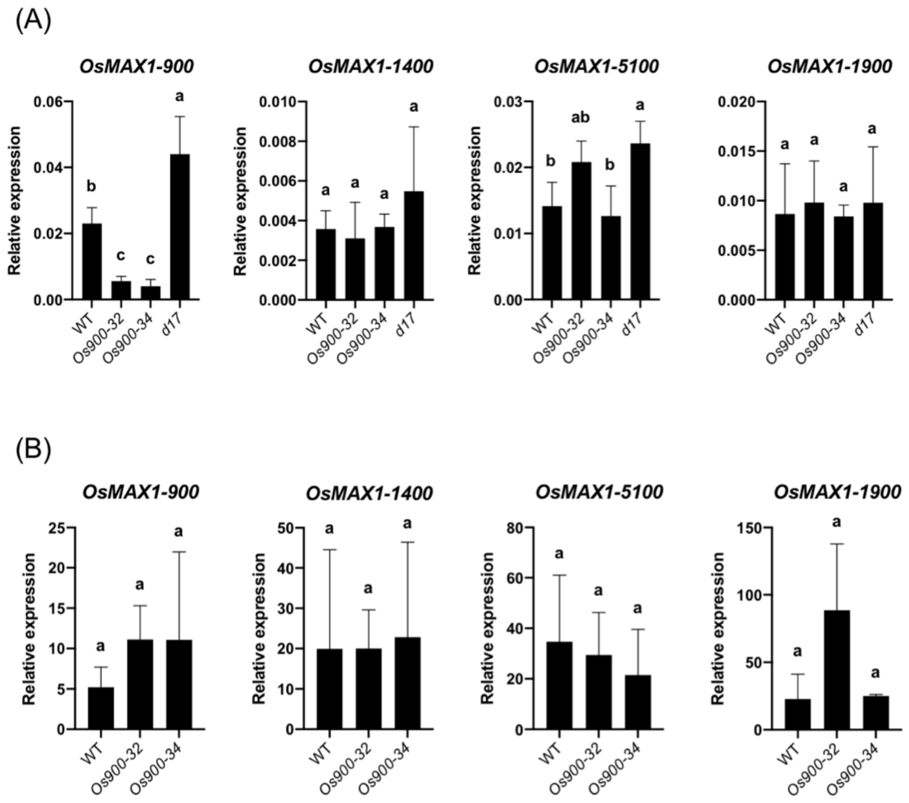

Fig. S5.

Transcript analysis of the SL biosynthetic genes in root tissues under (A) normal condition and (B) phosphate deficiency. The data are presented as means ± SD from three biological samples. Means not sharing a letter differ significantly at $P_{0.05}$.

Abbreviations: *MAX1*, *More Axillary Growth 1*

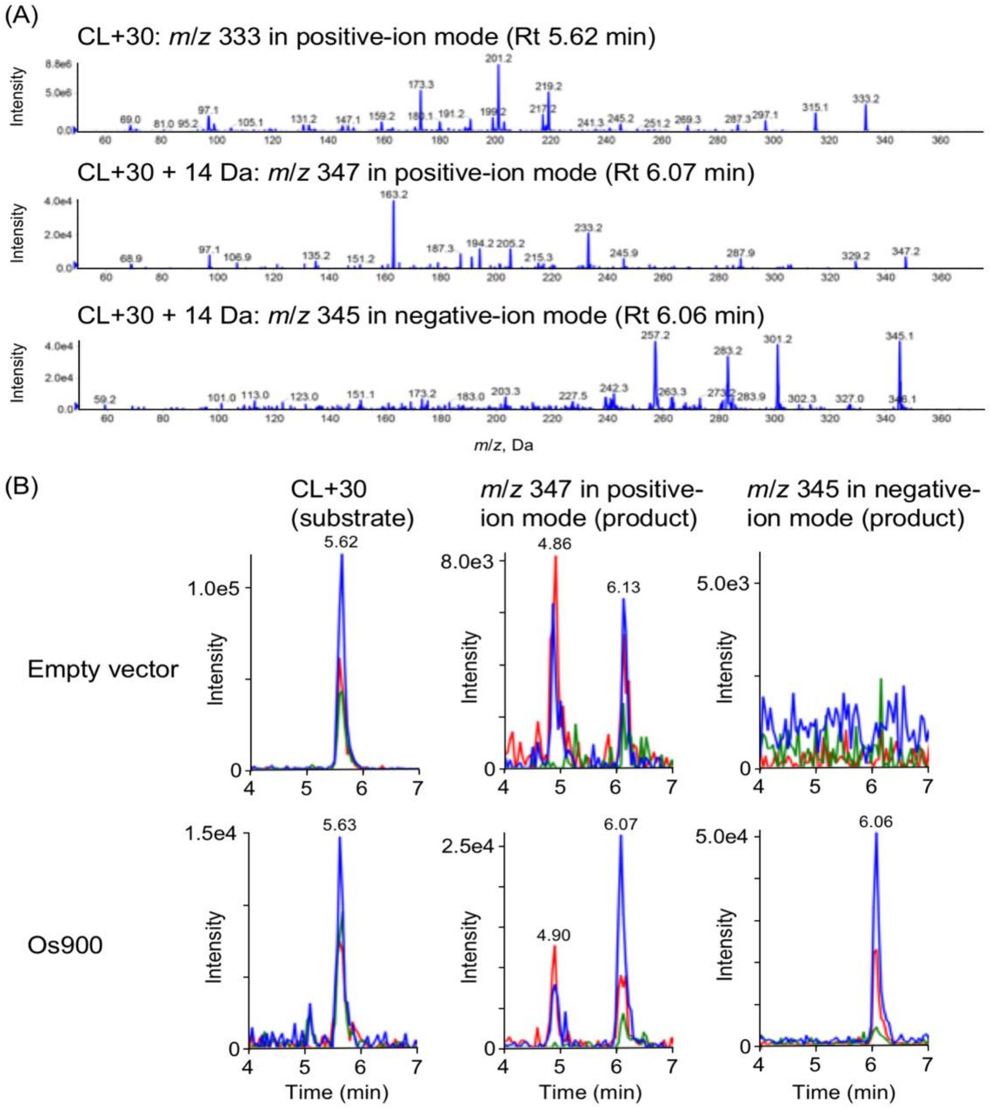

Fig. S6.
Enzymatic conversion of CL+30 containing fraction to CL+30+14 Da by Os900. (**A**) Product ion spectra derived from the precursor ion (*m*/*z* 333 $[M+H]^+$ in positive mode) of substrate CL+30 and the precursor ion (*m*/*z* 347 $[M+H]^+$ in positive mode and *m*/*z* 345 $[M-H]^-$ in negative mode) of CL+30 + 14 Da produced by Os900 are shown. (**B**) Multiple reaction monitoring chromatograms of CL+30 (blue, 333.20/97.00; red, 333.20/201.00; green, 333.20/219.00; *m/z* in positive mode), CL+30 + 14 Da (blue, 347.00/223.00; red, 347.00/97.00; green, 347.00/205.00; *m/z* in positive mode) and CL+30 + 14 Da (blue, 345.00/301.00; red, 345.00/257.00; green, 345.00/113.00; *m/z* in negative mode) are shown.

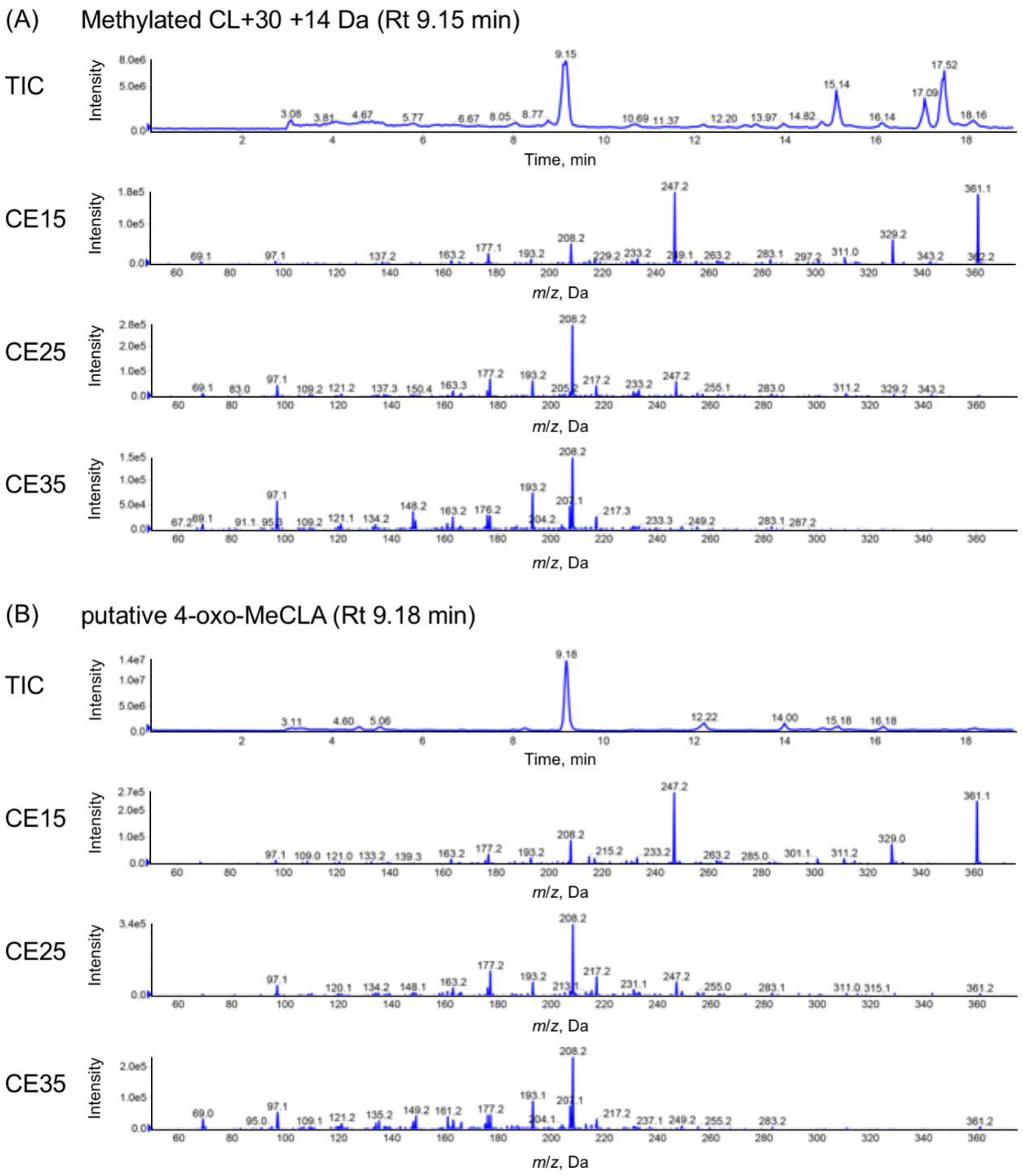

Fig. S7.

Product ion spectra of (A) methylated CL+30 + 14 Da and (B) putative 4-oxo-MeCLA in root exudates of rice *d14* mutant. Total ion chromatogram (TIC) and product ion spectra derived from the precursor ion of *m*/*z* 361 $[M+H]^+$ in positive mode by Collision Energy (CE) 15, 25 and 35 V were shown.

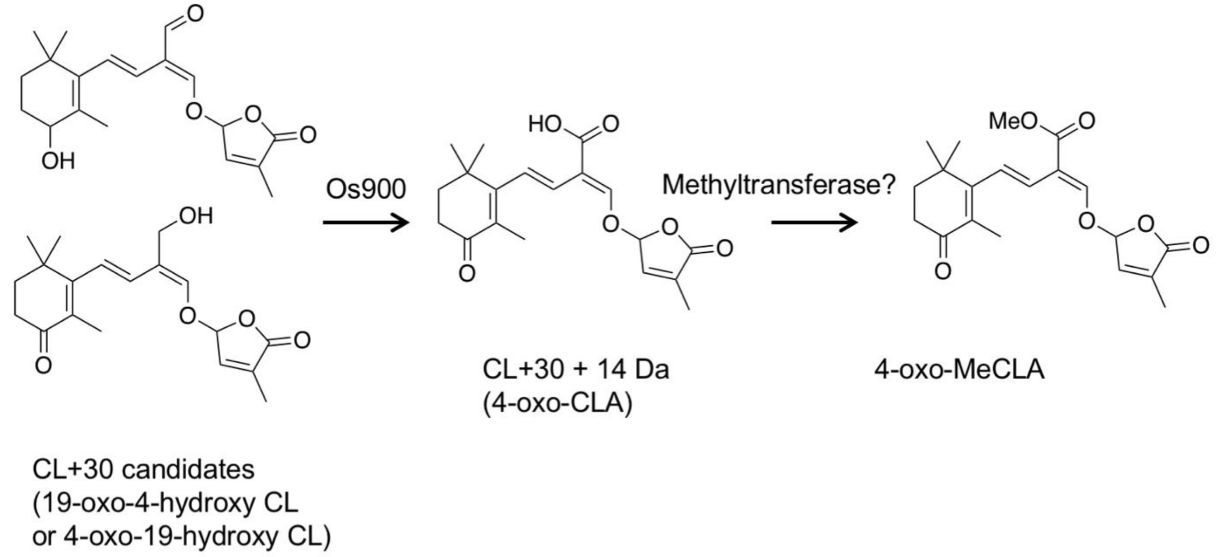

Fig. S8.
Proposed 4-oxo-MeCLA biosynthesis. Oxidation at C4 and C19 position by Os900 gives rise to 4-oxo-CLA. The conversion of 4-oxo-CLA to tentative 4-oxo-MeCLA is catalyzed by an unknown methyltransferase.

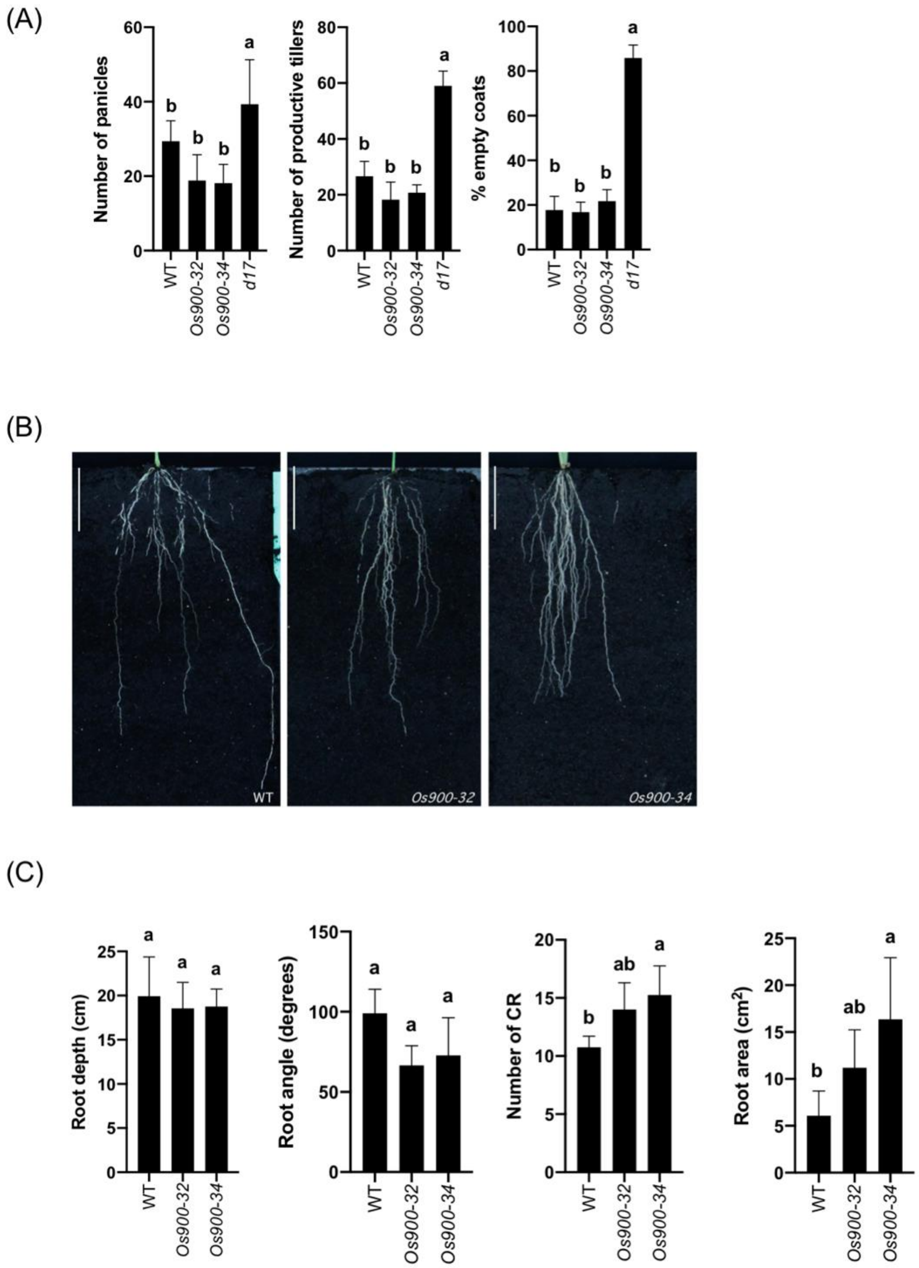

Fig. S9.
Phenotypes of *Os900*-KO lines. (**A**) Shoot phenotypes of WT, *Os900KO*-lines, and *d17* mutant plants grown in soil. The data are represented as mean ± SD from a number of biological replicates (5≤n≤7 for WT, *Os900-32* and *-34*, n=3 for *d17*). (**B** and **C**) Root phenotypes of WT and *Os900*-KO lines. The data are represented as mean ± SD from a number of samples n=4. Scale bar = 5 cm. The statistical significance is determined by one-way ANOVA and Tukey's multiple comparison test. Abbreviations: CR, crown roots.

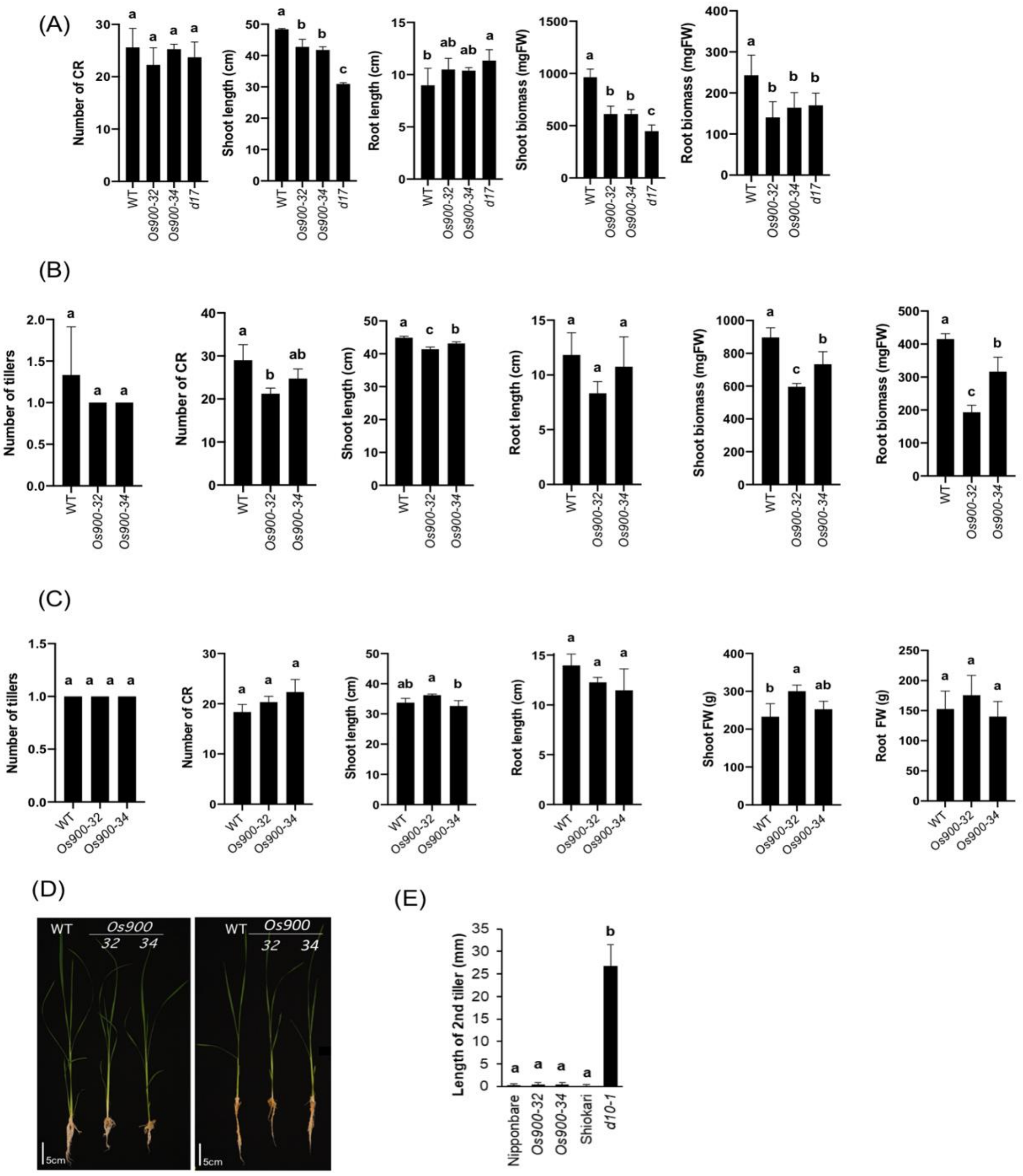

Fig. S10.

Phenotypes of *Os900*-KO lines grown in hydroponic culture under (A) +P, (B) –P, and (C) low Pi conditions. (**D**) Pictures of 3-week old seedlings grown under –Pi (left) and low Pi conditions (right). (**E**) Second tiller of two-week-old rice SL biosynthetic mutants *Os900*-KO lines 32 and 34, and *d10-1*. The data are presented as mean ± SD for the number of biological replicates (**A**, n=5 for WT, n=8 for *Os900-32*, n=4 for *Os900-34*, and n=7 for *d17*; **B** and **C**, 3≤n≤5; E, n=10). Scale bar = 5 cm. The statistical significance is determined by one-way ANOVA and Tukey's multiple comparison test. Abbreviations: CR, crown roots.

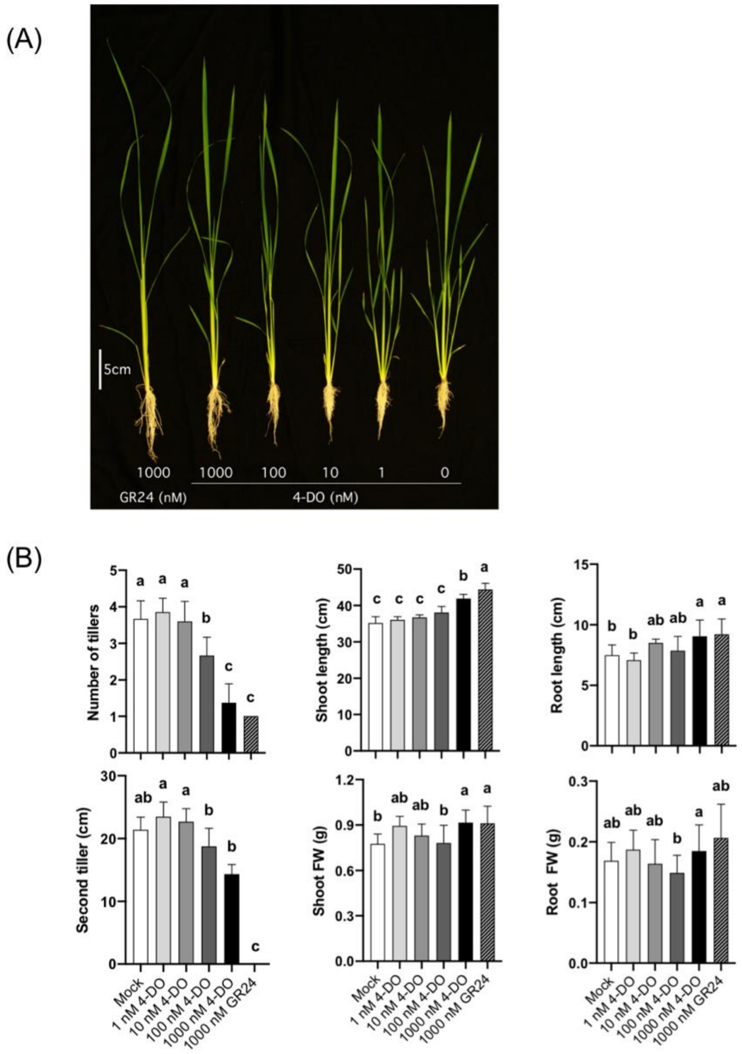

Fig. S11.

Effect of 4-deoxyorobanchol (4-DO) treatment on tillerng, shoot and root growth of *d17* SL deficient mutant. The values are represented as the mean ± SD number of biological replicates (5≤n≤9). Scale bar = 5 cm. The statistical significance is determined by one-way ANOVA and Tukey’s multiple comparison test.

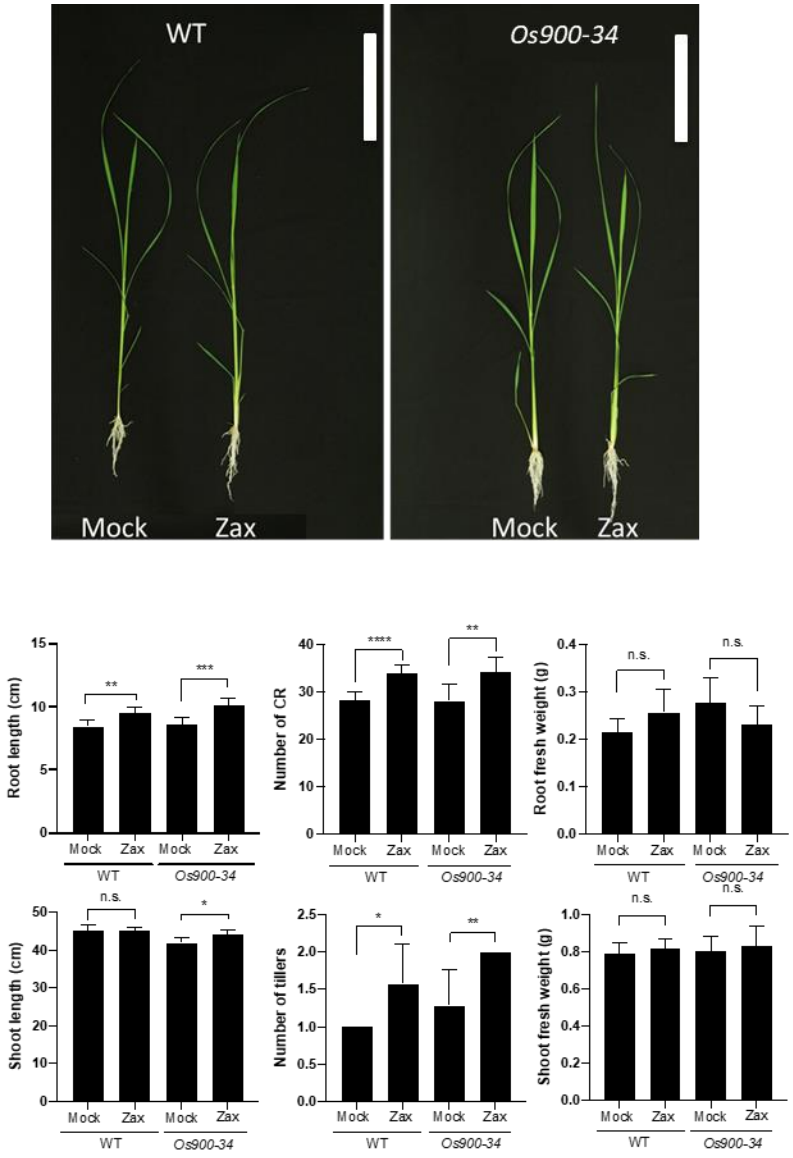

Fig. S12.
Effects of 2.5 µM zaxinone (Zax) on the growth of *Os900*-KO, compared to WT. Data represent the mean ± SD of 7 biological replicates. Scale bar = 15 cm. Statistical analysis was performed using one-way analysis of variance (ANOVA) and Tukey's post hoc test. (*P<0.05, ***P* < 0.01, ****P* < 0.001, *****P* ≤ 0.0001, n.s. not significant). Abbreviations: CR, crown roots.

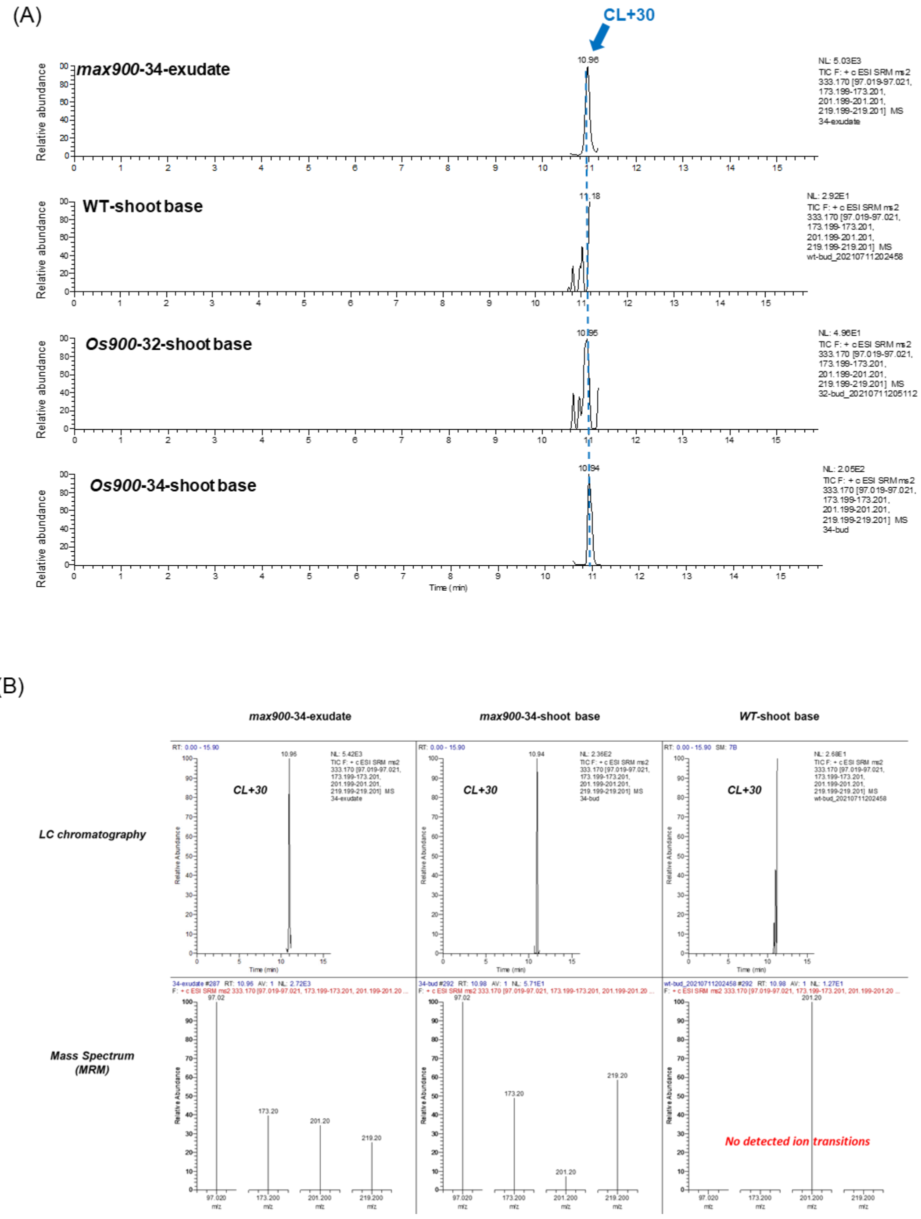

Fig. S13.

Detection of CL+30 (4-oxo-hydroxyl-CL) in *Os900-34* root exudates and in shoot base of *Os900-32* and *-34* plants grown under low Pi conditions. (**A**) (From up to down) Chromatograms of CL+30 detected in *Os900-34* root exudates, as well as in *Os900-32* and *-34* shoot base of plants grown in low Pi condition. CL+30 was not detectable in the shoot base of WT plants. (**B**) Representative CL+30 identification by multiple reaction monitoring (MRM) of four *m/z* fragmentations, including the diagnostic *m/z* 97.02 (D-ring). The shoot base samples were obtained from a pool of 12 shoot bases of each genotype grown under low Pi conditions. The blue arrows indicate the elution peaks of CL+30 with a retention time of 10.94 min.

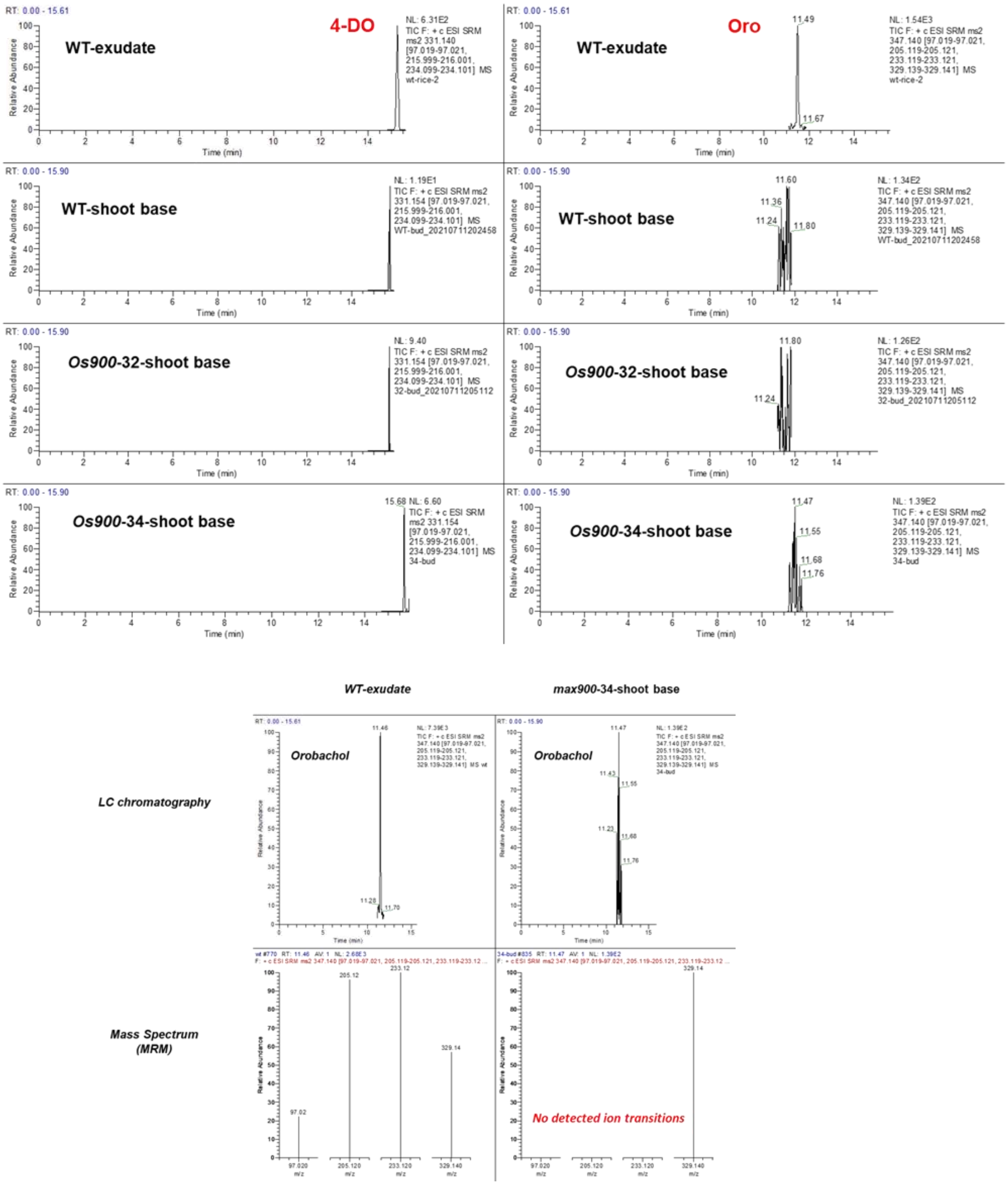

Fig. S14.

Detection of 4-DO (4-deoxyorobanchol) and Oro (orobanchol) in exudates of WT and shoot bases of WT, *Os900-32* and -*34* (low Pi condition). (**A**) Neither 4-DO nor orobanchol were detectable in shoot bases of WT or *Os900* plants. (**B**) Representative orobanchol identification by multiple reaction monitoring (MRM) of four *m/z* fragmentations, including the diagnostic *m/z* 97.02 (D-ring). The shoot base samples were obtained from a pool of 12 shoot bases of each genotype grown under low Pi conditions.

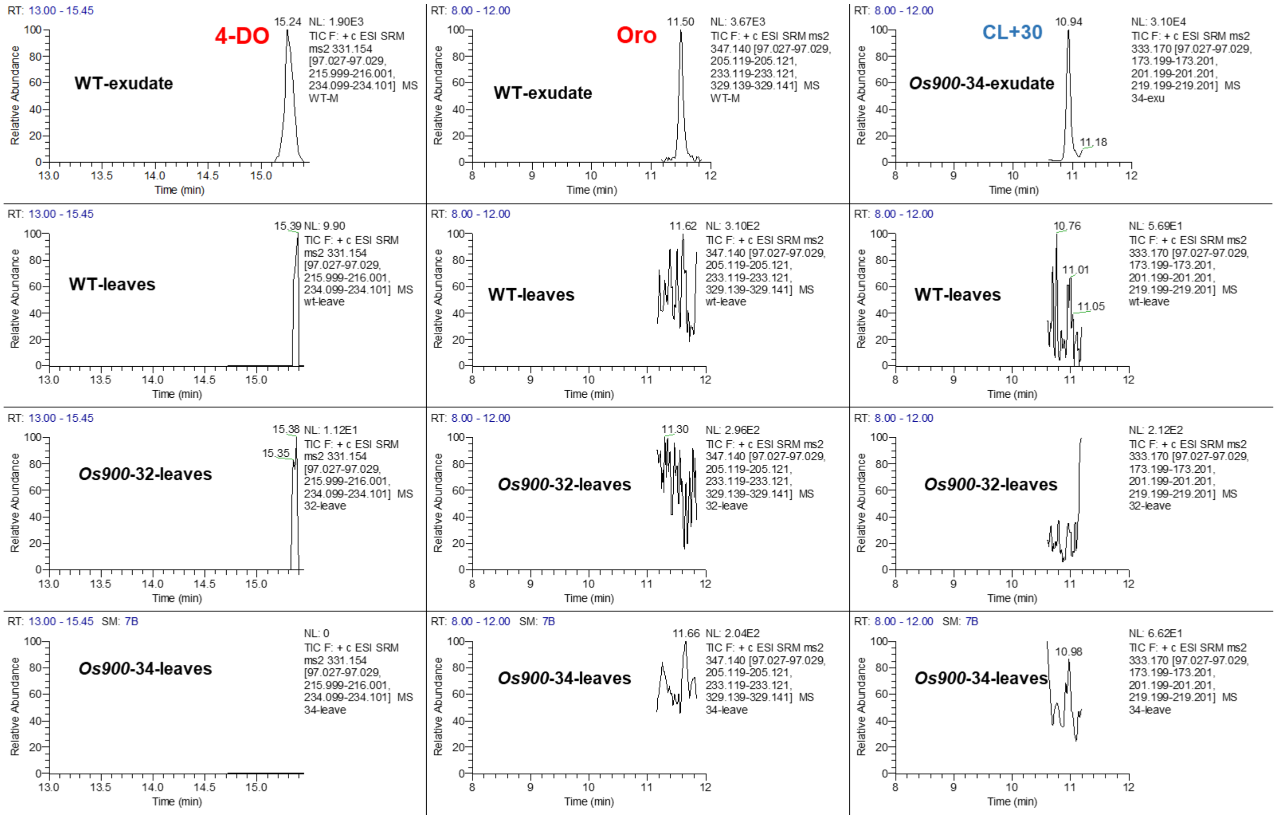

Fig. S15.

Detection of 4-DO (4-deoxyorobanchol), Oro (orobanchol), and CL+30 (4-oxo-hydroxyl-CL) in WT, *Os900-32* and *-34* leaves (low Pi condition). SL leaf extracts were directly compared with the WT and *Os900-34* root exudates that were used as natural standards. None of the SLs detected in exudates was found in the leaves. The samples were obtained from a pool of 16 biological replicates of each genotype grown under low Pi conditions.

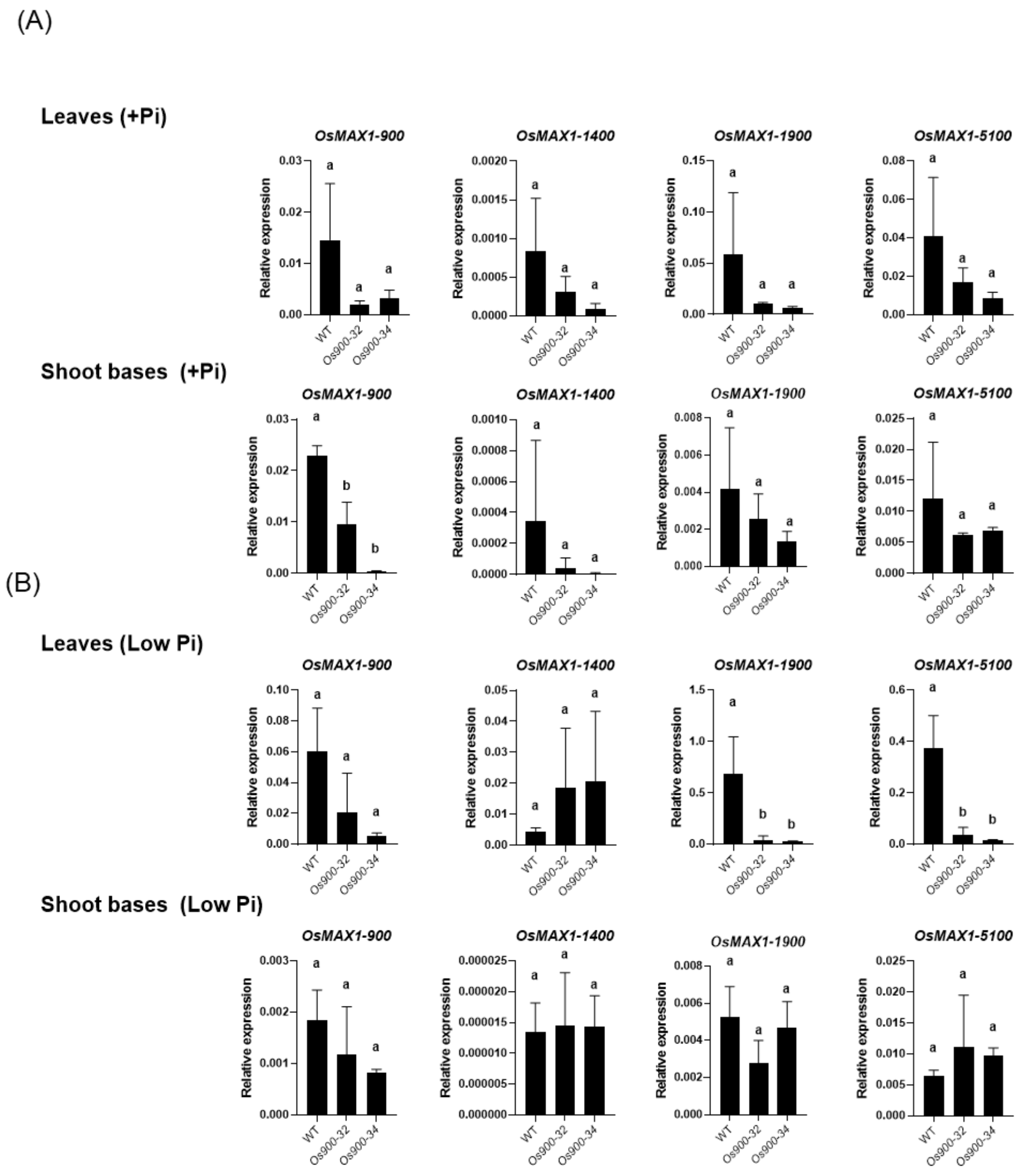

Fig. S16.

Transcript analysis of the rice *MAX1* homologs in shoot bases and leaf tissues under (A) normal condition and (B) phosphate deficiency. The data are presented as means ± SD from three biological samples. Each biological replicates contained 5 to 6 individual plants. Means not sharing a letter differ significantly at $P_{0.05}$.

Abbreviations: *MAX1*, *More Axillary Growth 1*

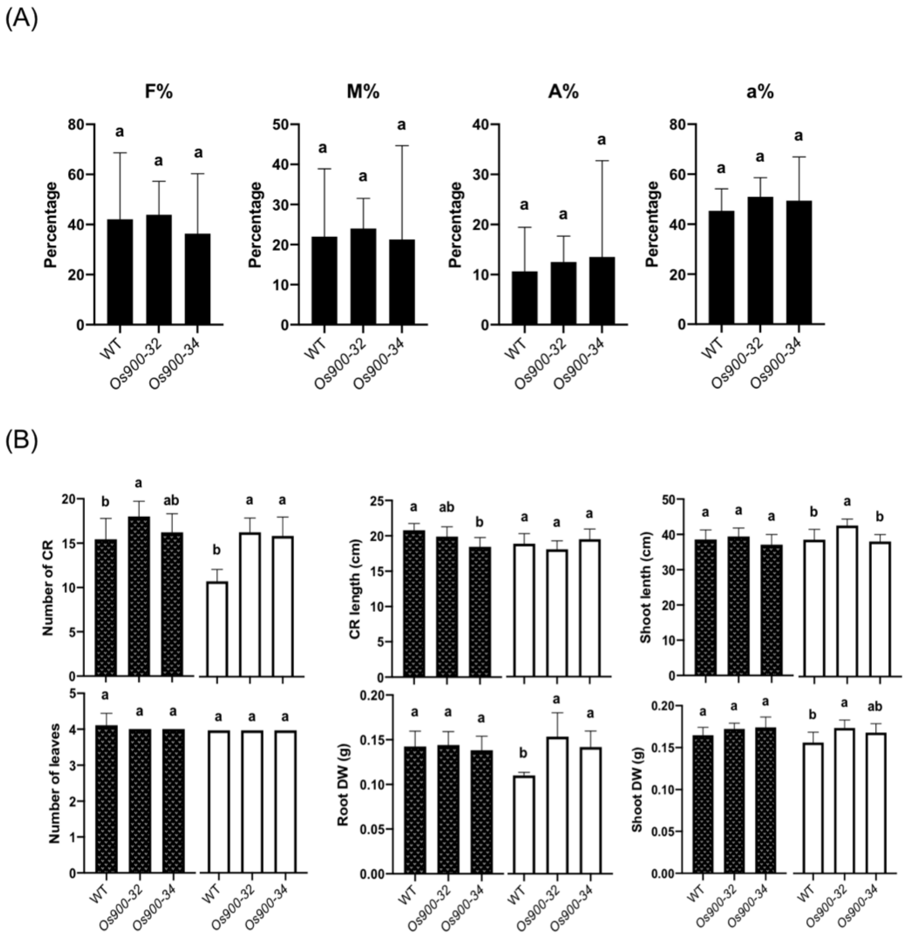

Fig. S17.

Evaluation of AM symbiosis colonization in *Osmax1* mutant lines. (**A**) Mycorrhizal colonization of WT and *Os900*-KO lines by the AM fungus *Rhizophagus irregularis* at 35 dpi. Degree of colonization expressed as mycorrhizal frequency (F %), intensity (M %) and arbuscule abundance (A %); with the percentage of arbuscules within infected (a%) in the root system of WT and *Os900*-KO lines. Data are represented as the mean ± SD for 4 plants. (**B**) Plant phenotype comparison with (plain bars) and without (white bars) mycorrhizal colonization by *R. irregularis* at 35 dpi for WT and *Os900*-KO lines. The collected traits were number of crown roots (CR), CR and shoot length, number of leaves, root and shoot dry weight (DW). The data are represented as the mean ± SD of 9 plants grown with AM fungi and 7 plants grown without. The statistical significance is determined by one-way ANOVA and Tukey's multiple comparison test.

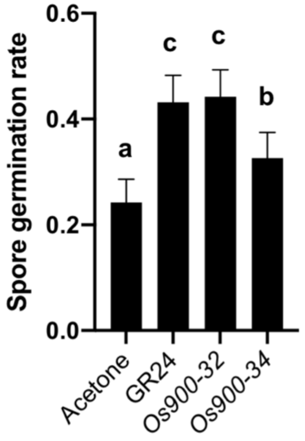

Fig. S18.
Evaluation of the effect of *Os900*-KO exudates on *Gigaspora margarita* spore germination. Acetone (Mock), GR24, and root exudates of *Os900-32* and *-34* were applied on *G. margarita* spores, and spore germination was reported. Data are the average of 96 biological replicates ± SD.

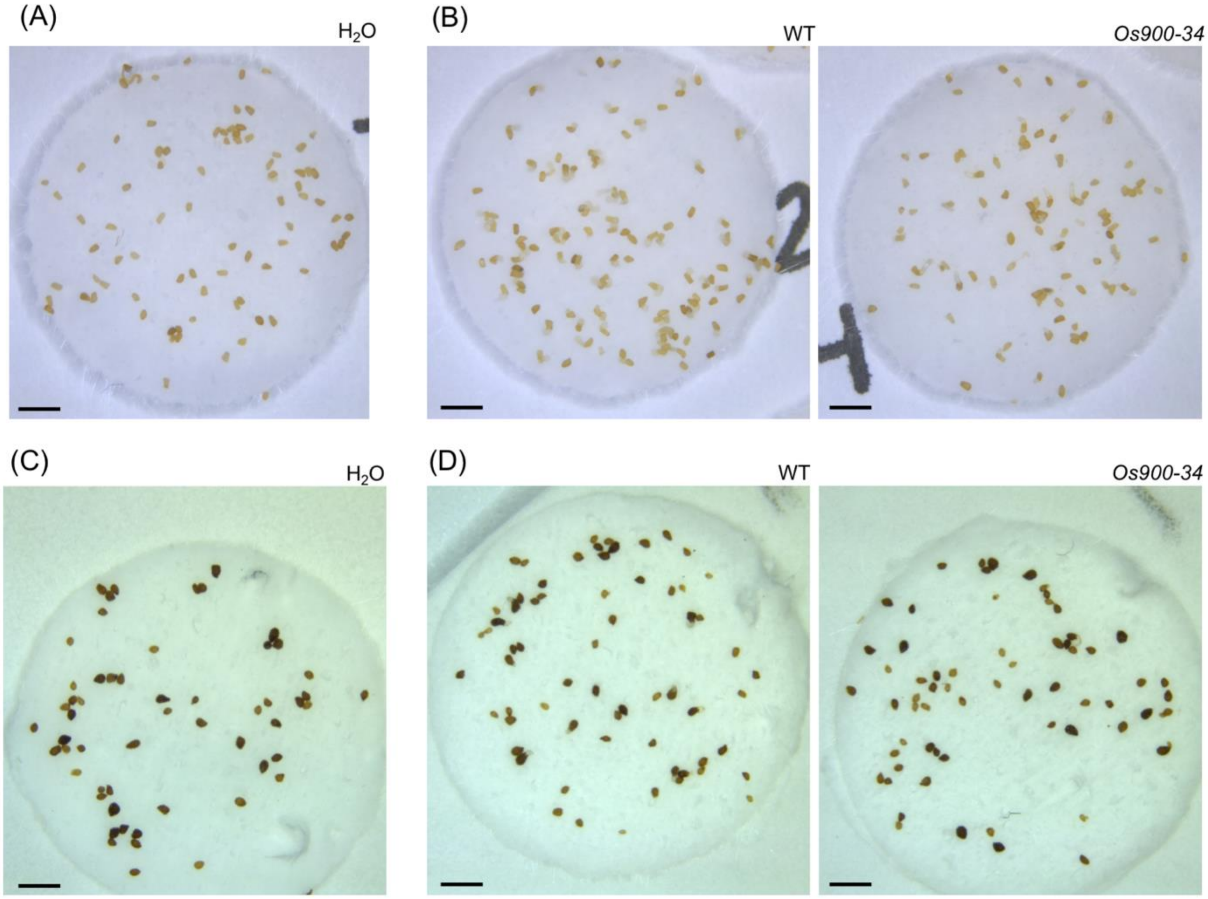

Fig. S19.
Seed germination of root parasitic weeds by treatment with root exudates. (**A**-**B**) *Striga hermonthica* and (**C**-**D**) *Phelipanche ramose* seeds. Scale bar = 100 µm.

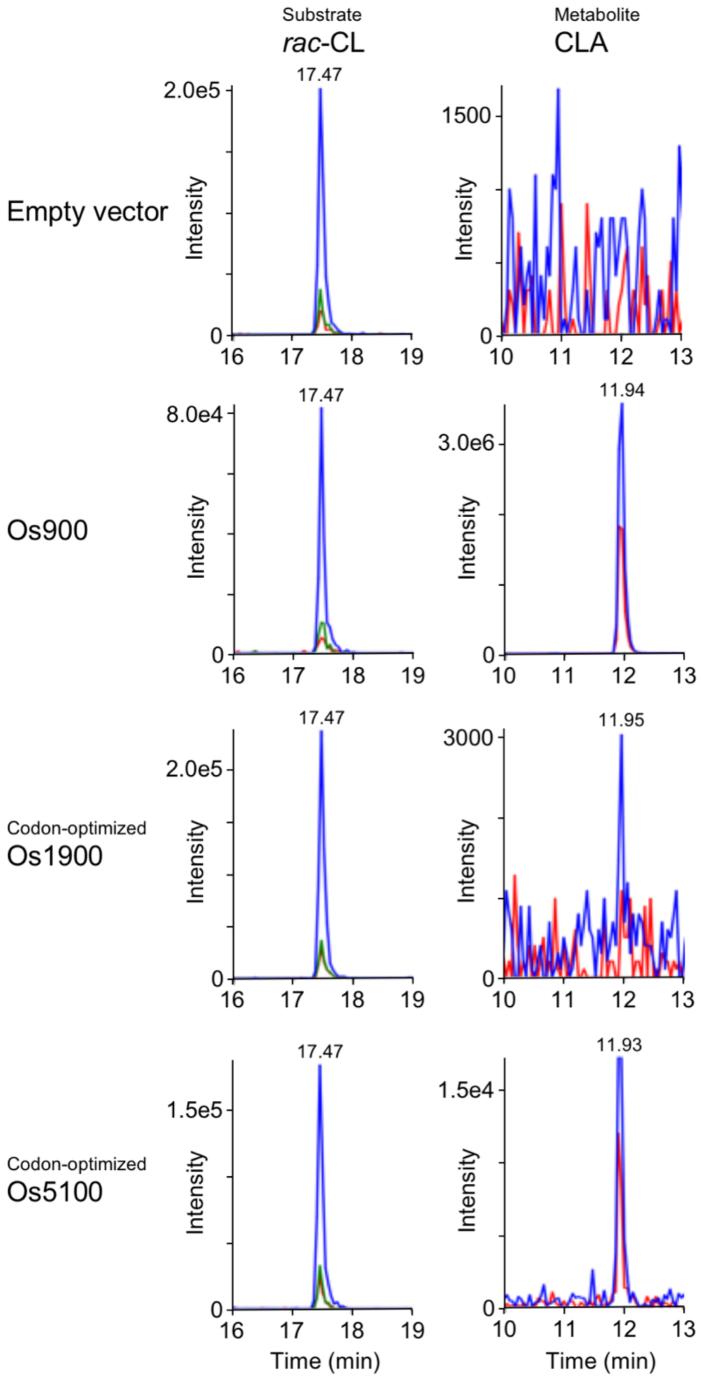

Fig. S20.
Enzymatic conversion of CL into CLA by rice MAX1 homologs. Multiple reaction monitoring chromatograms of CL (blue, 303.00/97.00; red, 303.00/189.00; green, 303.00/207.00; *m/z* in positive mode) and CLA (blue, 331/113; red, 331/69; *m/z* in negative mode) are shown.

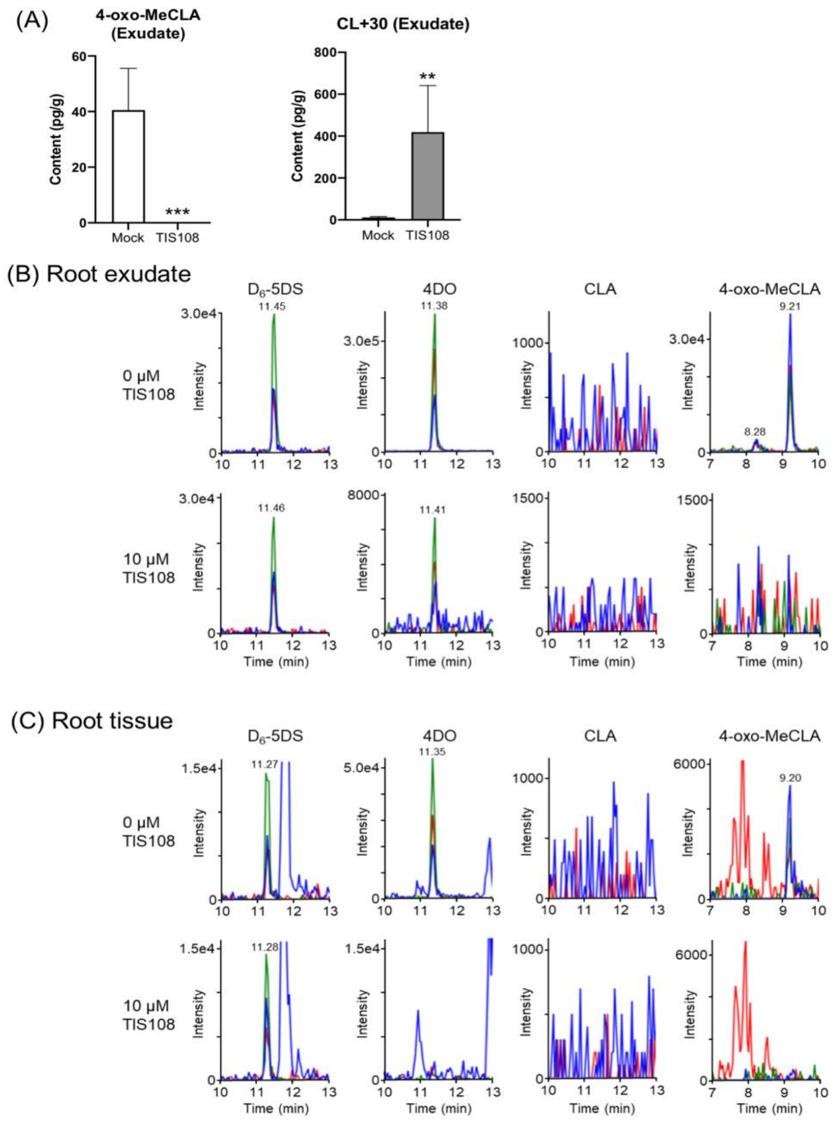

Fig. S21.

Quantification of SLs in root exudates (A-B) and tissues (C) of wild-type rice. Multiple reaction monitoring chromatograms of D_6-5DS (blue, 337/97; red, 337/222; green, 337/240; *m/z* in positive mode), 4DO (blue, 331/97; red, 331/216; green, 331/234; *m/z* in positive mode), CLA (blue, 331/113; red, 331/69; *m/z* in negative mode) and putative 4-oxo-MeCLA (blue, 361/208; red, 361/177; green, 361/97; *m/z* in positive mode) are shown. The data are presented as means ± SD of 5 biological replicates. Asterisk indicates significant difference without (Mock) and with 10 μM TIS108 treatment (TIS108) (***P*<0.01, ****P*<0.001, Student's t test).

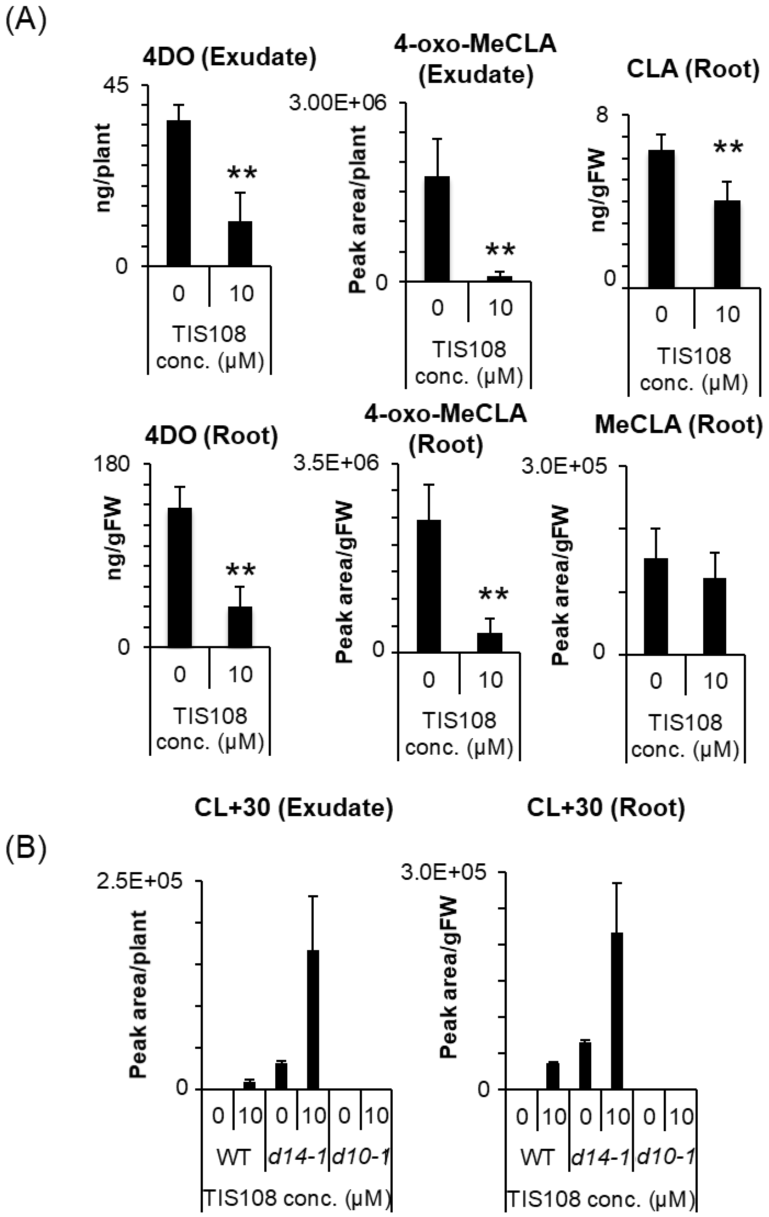

Fig. S22.

Effects of TIS108 on SL level of *d14-1* rice. (**A**) Endogenous levels of SLs in roots and root exudates of the *d14-1* mutant. The data are presented as means ± SD (n = 4). ** means statistically different from that of 0 μM TIS108 rice (t test, $P < 0.01$). (**B**) Endogenous levels of CL+30 in root exudates (left) and roots (right). The data are presented as means ± SD (n = 3).

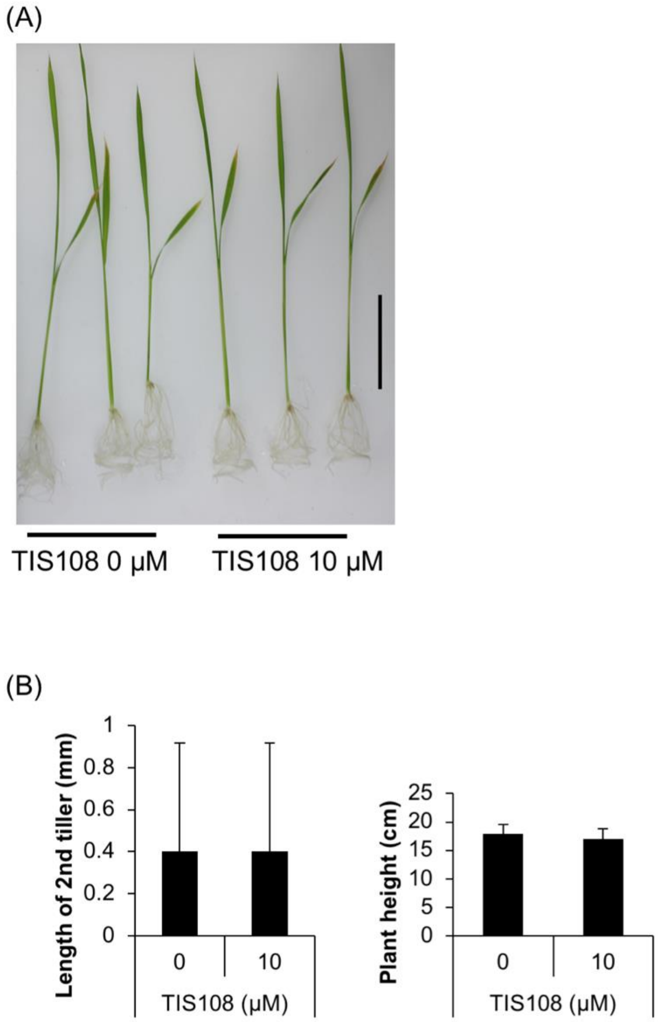

Fig. S23.
Effect of TIS108 treatment (10 μM) on two-week-old rice seedlings. (**A**) Phenotype of hydroponically grown seedlings. Scale bar = 5 cm. (**B**) Second tiller length (upper) and plant height (lower). The data are presented as means ± SD from 10 samples.

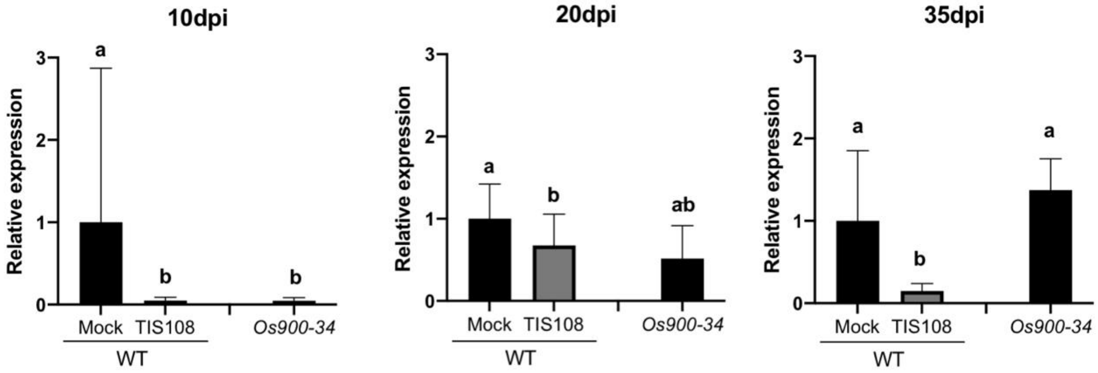

Fig. S24.
Effect of TIS108 on mycorrhization. AM colonization was quantified by measuring the expression of *OsPT11* plant marker gene. The data are represented as the mean ± SD of number of samples n (n=6). The statistical significance is determined by Kruskal Wallis' test (non-parametric test).

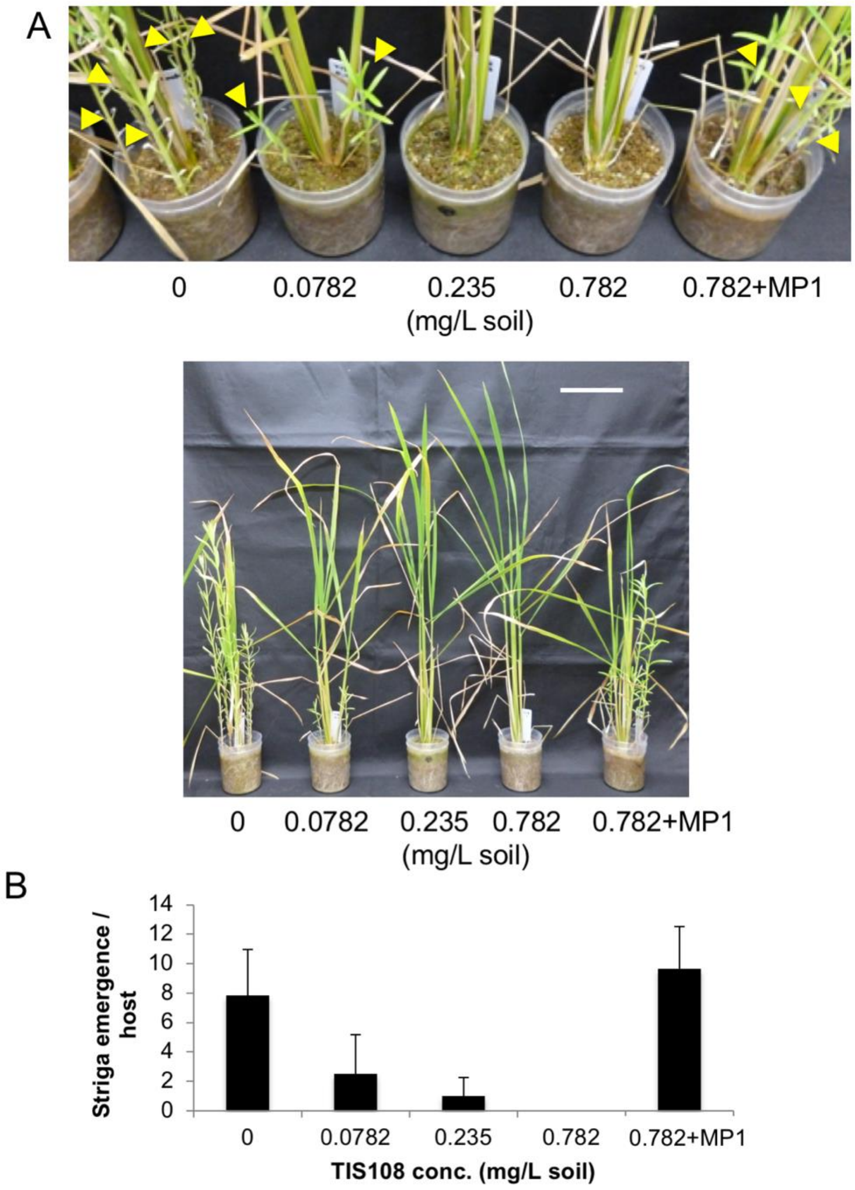

Fig. S25.
***Striga* emergence test.** Rice plants were co-cultivated with *Striga* for 7 weeks. The soil was weekly treated with acetone or TIS108. (**A**) Arrowheads indicate emerged *Striga*. Scale bar indicate 10 cm. (**B**) Number of emerged *Striga* after 7 weeks of co-cultivation. The data are means ± SD of 6 samples.

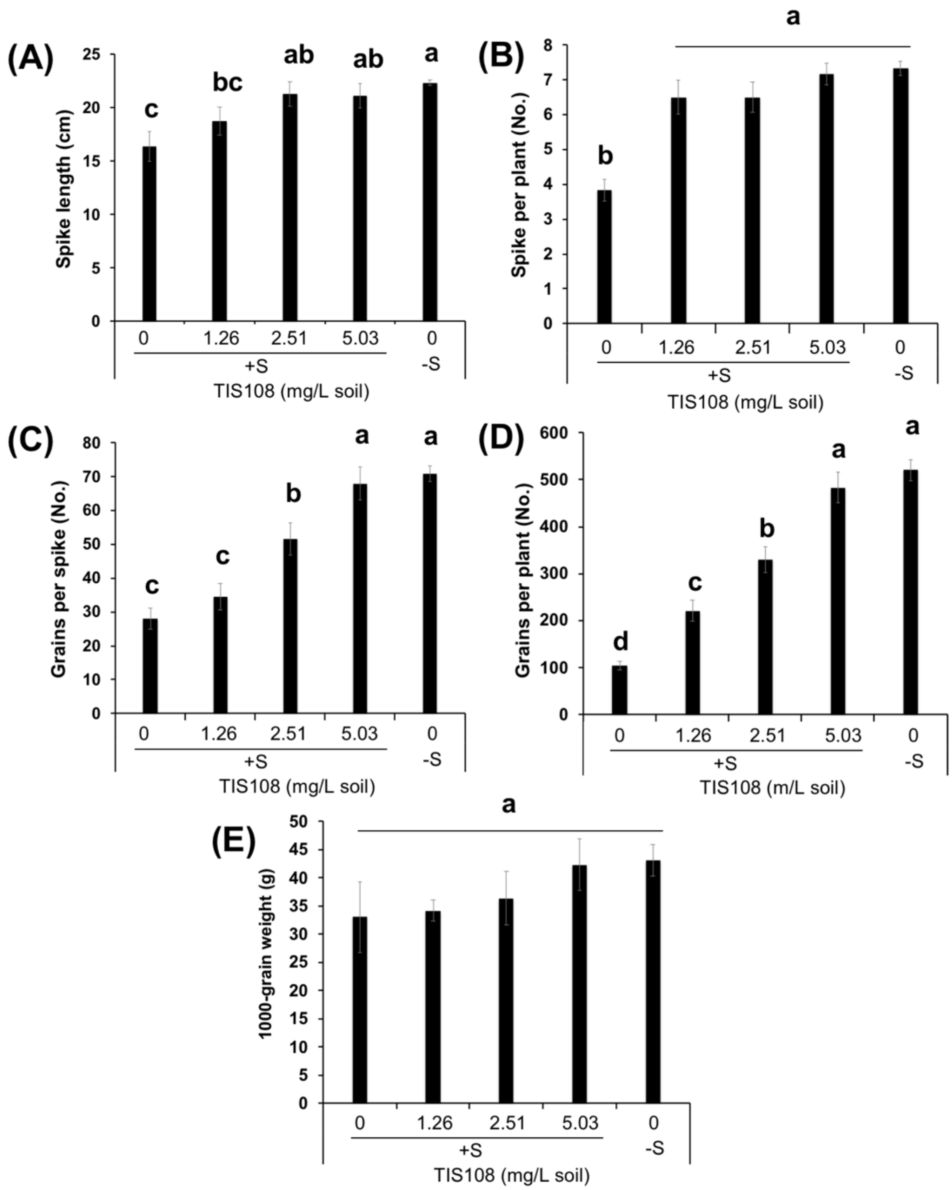

Fig. S26.
Effects of TIS108 on rice growth and yield grown in soil with (+S) or without (-S) *Striga* seeds. The soil was treated by TIS108 for three weeks at one-week interval. Spike length (**A**), number of spike (**B**), grains per spike (**C**), grains per plants (**D**), and 1000 grain weight (**E**) were recorded at the time of final harvesting. The data means ± SE of 6 samples. Means not sharing a letter in common differ significantly at $P_{0.05}$.

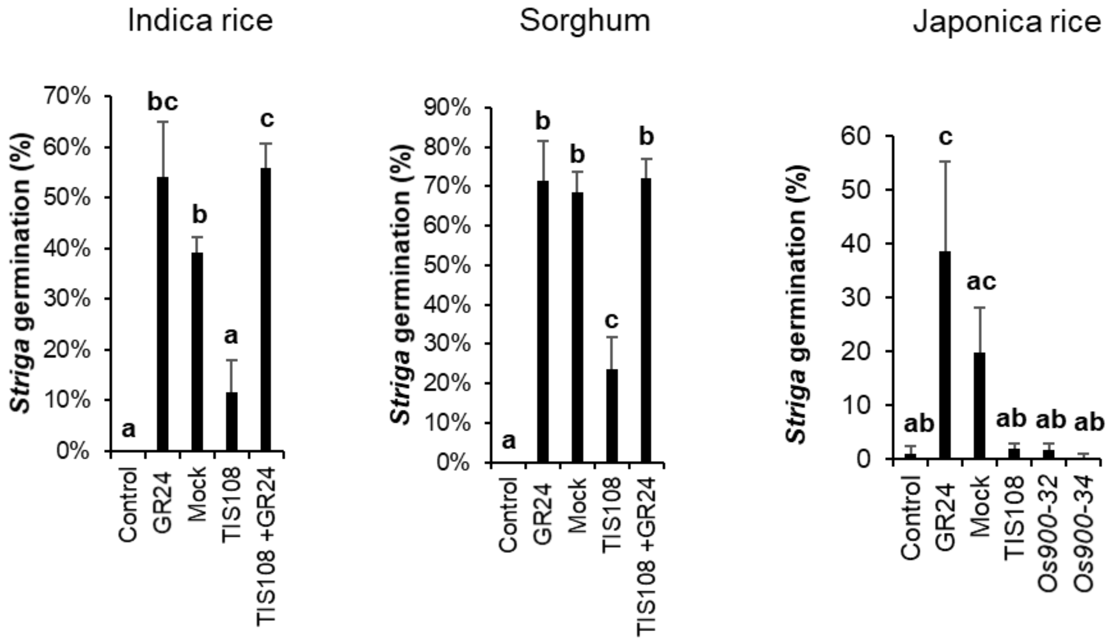

Fig. S27.
***Striga* germination test of root exudates of TIS108-treated Indica rice, sorghum, and Japonica rice.** Control: water, GR24: 1 μM *rac*-GR24, Mock: Root exudates of mock-treated plants, TIS108: Root exudates of 10 μM TIS108-treated plants, TIS108+GR24: Root exudates of 10 μM TIS108-treated plants and 1 μM *rac*-GR24. The data are presented as means ± SD from 3-5 biological samples. Different letters indicate statistically significant differences at *P*0.05.

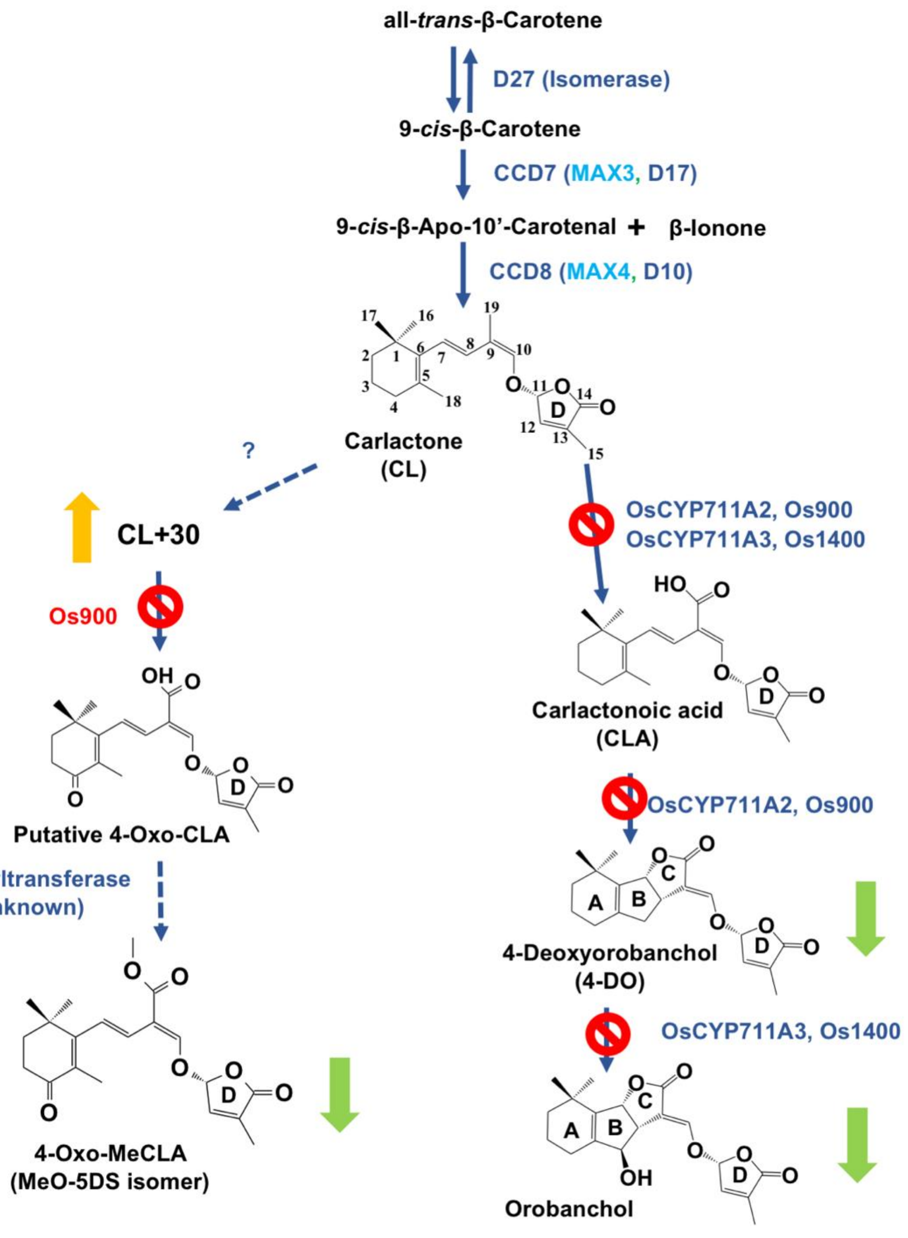

Fig. S28.
TIS108, a MAX1-specific inhibitor of SL biosynthesis. Abbreviations: D, Dwarf; CCD, Carotenoid Cleavage Dioxygenase; MAX, More Axillary Growth; Os900, OsMAX1-900; Os1400, OsMAX1-1400; CYP, Cytochrome P450; MeO-5DS, Methoxy-5-Deoxy-Strigol.

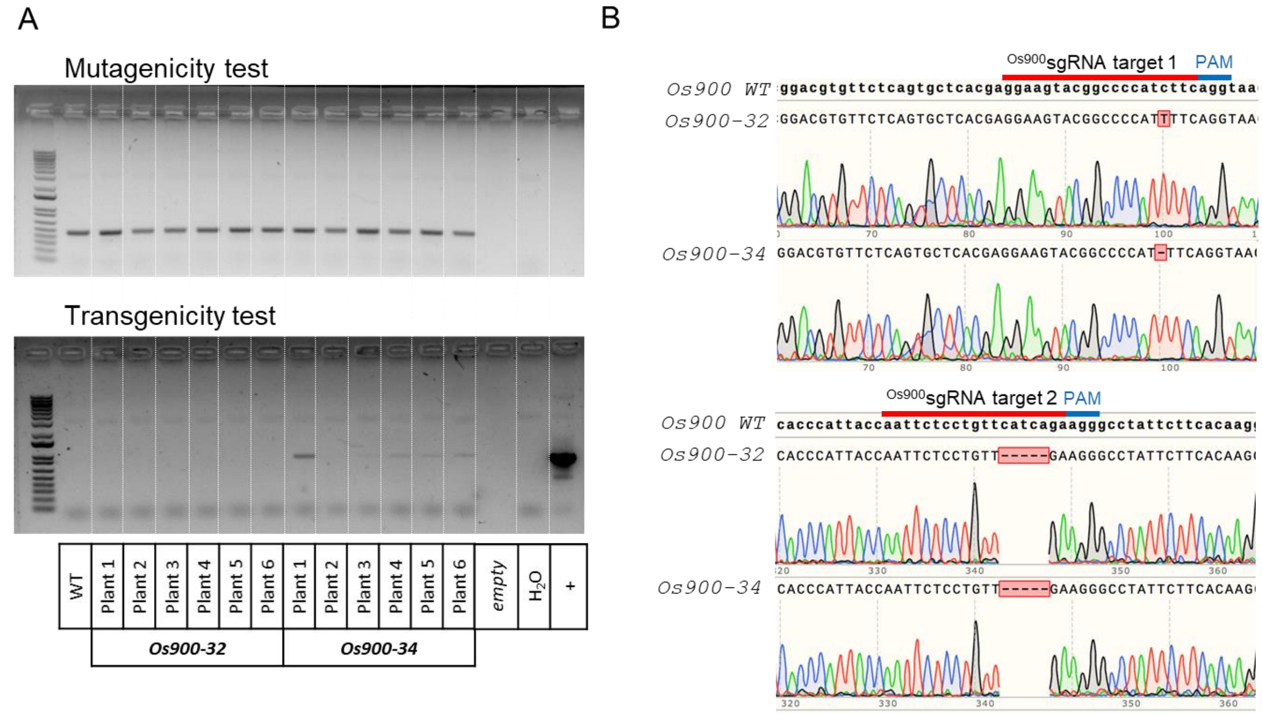

Fig. S29.

Genotyping of Os*900*-KO lines. (**A**) Genomic DNA amplification of the region surrounding sgRNA target site in wild-type (WT) and *Os900*-KO lines 32 and 34 (6 plants each) (up, mutagenicity test) and pRGEB32 region containing the two *Os900*sgRNAs sequences (down, transgenicity test). Water (H_2O) and the pRGEB32 vector containing the two *Os900*sgRNAs sequences were used as a negative and positive (+) control, respectively. (**B**) Sequencing details of two representative plants of the homozygous *Os900*-KO lines showing the different mutations present in each line, aligned to the WT sequence for both *Os900* sgRNAs target sites.

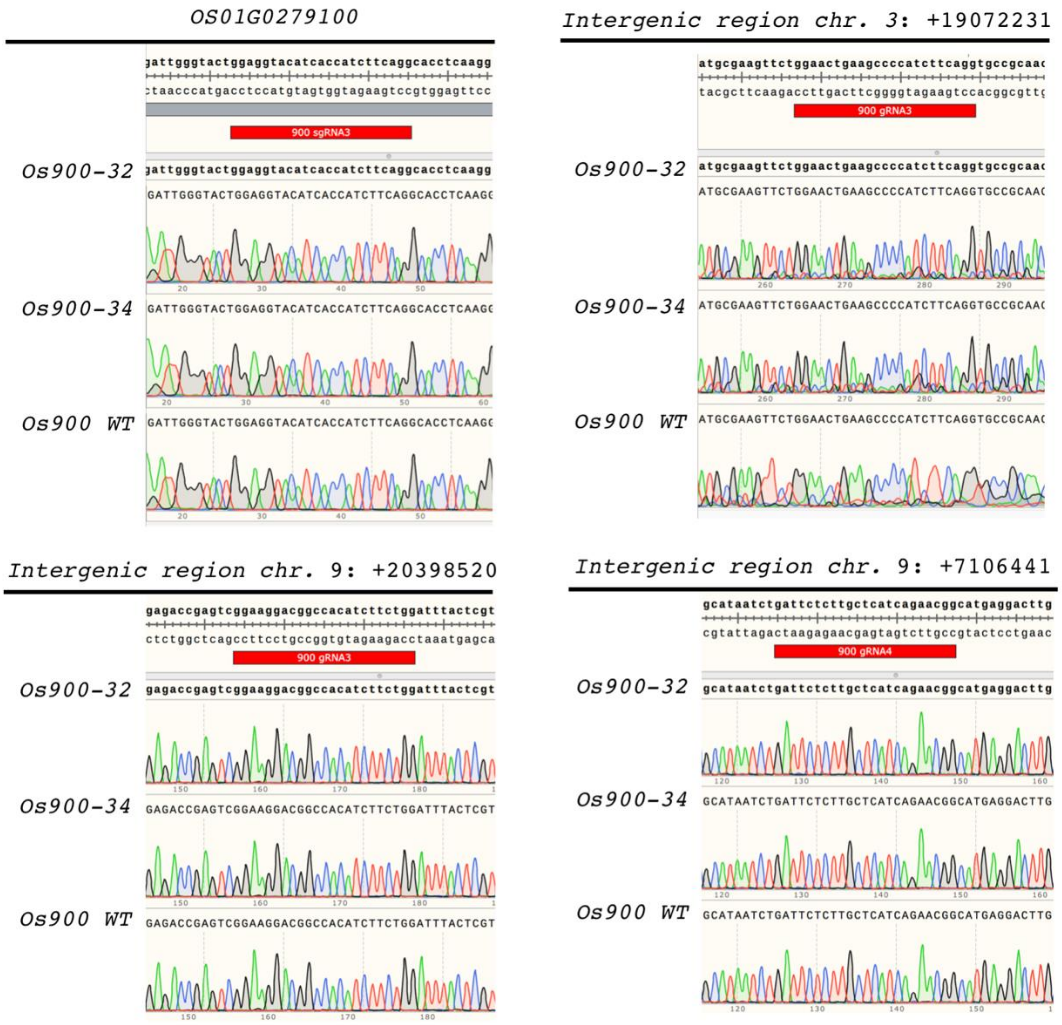

Fig. S30.

Off-target studies of Os*900* sgRNA3 and sgRNA 4. Sequencing details of two representative plants of the homozygous *Os900*-KO lines showing that 4 predicted off-target sites are not mutated. These sequences are aligned to the consensus sequence and WT sequence. The off-target sequences were predicted using the online tool CRISPR-P 2.0 (http://cbi.hzau.edu.cn/cgi-bin/CRISPR2/CRISPR), using *Oryza sativa* (RAP-DB) as a target genome and 400bp surrounding the studied gRNA target site as an entry sequence. See table S5 for the primer sequences.

Table S1. Genes up-regulated in 10 μM TIS108-treated rice

Table S1 Up-regulated genes of 10 μM TIS108-treated rice				
Gene Symbol	RAP ID	MSU ID	Description	Fold change
gene:Os04g0649700	Os04g0649700	LOC_Os04g55620.1	receptor kinase, putative, expressed	731.74
gene:Os03g0784800	Os03g0784800	LOC_Os03g57130.1	expressed protein	592.28
OsUMAMIT6	Os02g0703900	LOC_Os02g47500.1	nodulin, putative, expressed	464.19
gene:Os05g0102800	Os05g0102800	LOC_Os05g01240.1	AML1, putative, expressed	80.88
gene:Os02g0519950	Os02g0519950	None		69.14
gene:Os10g0361000	Os10g0361000	LOC_Os10g21670.1	dehydration stress-induced protein, putative, expressed	64.29
OsLAC8	Os01g0850700	LOC_Os01g63190.1	laccase precursor protein, putative, expressed	57.93
gene:Os11g0137300	Os11g0137300	LOC_Os11g04220.1	HEAT repeat family protein, putative, expressed	39.23
Cen8.t01003	Os08g0314800	LOC_Os08g22354.1	polyadenylate-binding protein, putative, expressed	37.24
gene:Os03g0191100	Os03g0191100	LOC_Os03g09110.1	mitochondrial carrier protein, putative, expressed	21.51
gene:Os01g0237750	Os01g0237750	LOC_Os01g13610.1	isoflavone reductase homolog IRL, putative, expressed	20.49
OsPDR9	Os01g0609300	LOC_Os01g42380.1	pleiotropic drug resistance protein, putative, expressed	19.60
gene:Os01g0760701	Os01g0760701	None		19.54
OsPR10a	Os12g0555500	LOC_Os12g36880.1	pathogenesis-related Bet v I family protein, putative, expressed	18.62
OsABCG35	Os01g0609200	LOC_Os01g42370.1	pleiotropic drug resistance protein, putative, expressed	18.49
gene:Os01g0237500	Os01g0237500	LOC_Os01g13610.1	isoflavone reductase homolog IRL, putative, expressed	17.51
SSIVA	Os01g0720600	LOC_Os01g52250.1	starch synthase, putative, expressed	17.36
OsPDR9	Os01g0609300	LOC_Os01g42380.1	pleiotropic drug resistance protein, putative, expressed	16.99
gene:Os01g0937012	Os01g0937012	None		15.20
OsAMT2_2	Os01g0831900	LOC_Os01g61550.1	ammonium transporter protein, putative, expressed	15.14
RAB21_2	Os11g0454000	LOC_Os11g26760.1	dehydrin, putative, expressed	13.70
OsLEA25	Os11g0451700	LOC_Os11g26570.1	dehydrin, putative, expressed	13.67
OsCYP709C9	Os07g0418500	LOC_Os07g23570.1	cytochrome P450 72A1, putative, expressed	13.66
RAB21_1	Os11g0453900	LOC_Os11g26750.1	dehydrin, putative, expressed	12.25
gene:Os01g0609501	Os01g0609501	None		12.18
RAB16B	Os11g0454200	LOC_Os11g26780.1	dehydrin, putative, expressed	11.14
gene:Os11g0311300	Os11g0311300	LOC_Os11g20689.1	exosome complex exonuclease, putative, expressed	9.47
gene:Os05g0513900	Os05g0513900	LOC_Os05g43830.1	hydrolase, alpha/beta fold family domain containing protein, expressed	9.36
gene:Os05g0410601	Os05g0410601	None		9.10
OsEnS-53	Os03g0723400	LOC_Os03g51350.1	expressed protein	8.84
Oschib1	Os10g0416500	LOC_Os10g28080.1	glycosyl hydrolase, putative, expressed	8.62
gene:Os05g0151200	Os05g0151200	LOC_Os05g05930.1	peripheral-type benzodiazepine receptor, putative, expressed	8.29
OsLEA16	Os03g0168100	LOC_Os03g07180.1	embryonic protein DC-8, putative, expressed	6.91
Rab16A	Os11g0454300	LOC_Os11g26790.1	dehydrin, putative, expressed	6.77
gene:Os06g0691700	Os06g0691700	None		6.76
OsMaT-2	Os02g0483500	LOC_Os02g28170.1	transferase family protein, putative, expressed	6.52
gene:Os03g0429800	Os03g0429800	LOC_Os03g31550.1	aldehyde oxidase, putative, expressed	6.49
gene:Os03g0792850	Os03g0792850	None		6.35
RSOsPR10	Os12g0555000	LOC_Os12g36830.1	pathogenesis-related Bet v I family protein, putative, expressed	6.12
OsKMD3	Os02g0563000	LOC_Os02g35530.1	OsFBK8 - F-box domain and kelch repeat containing protein, expressed	5.92
gene:Os07g0585900	Os07g0585900	LOC_Os07g39720.1	expressed protein	5.89
OsTHI9	Os06g0514800	LOC_Os06g32240.1	THION9 - Plant thionin family protein precursor, expressed	5.66
gene:Os01g0639600	Os01g0639600	LOC_Os01g45250.1	DUF1645 domain containing protein, putative, expressed	5.48
OsGH3-7	Os06g0499500	LOC_Os06g30440.1	OsGH3.7 - Probable indole-3-acetic acid-amido synthetase, expressed	5.39
OsCCR10	Os02g0811800	LOC_Os02g56700.1	dehydrogenase, putative, expressed	5.31
gene:Os04g0653700	Os04g0653700	LOC_Os04g55980.1	glycine-rich RNA-binding, abscisic acid-inducible protein, putative, expressed	5.22
OsSub31	Os03g0761500	LOC_Os03g55350.1	OsSub31 - Putative Subtilisin homologue, expressed	5.14
gene:Os05g0526700	Os05g0526700	LOC_Os05g45070.1	harpin-induced protein 1 domain containing protein, expressed	5.14
gene:Os02g0207800	Os02g0207800	None		5.07
AWPM-19	Os05g0381400	LOC_Os05g31670.1	AWPM-19-like membrane family protein, putative, expressed	5.01
Cht6	Os02g0605900	LOC_Os02g39330.1	CHIT1 - Chitinase family protein precursor, expressed	4.97
ABCG43	Os07g0522500	LOC_Os07g33780.1	pleiotropic drug resistance protein 5, putative, expressed	4.85
SPL7	Os05g0530400	LOC_Os05g45410.1	HSF-type DNA-binding domain containing protein, expressed	4.83
gene:Os06g0129900	Os06g0129900	LOC_Os06g03930.1	cytochrome P450 86A1, putative, expressed	4.79
RPR10b	Os12g0555200	LOC_Os12g36850.1	pathogenesis-related Bet v I family protein, putative, expressed	4.49
gene:Os01g0838600	Os01g0838600	LOC_Os01g62130.1	ZOS1-14 - C2H2 zinc finger protein, expressed	4.42
gene:Os06g0239200	Os06g0239200	LOC_Os06g13190.1	expressed protein	4.39
gene:Os01g0644200	Os01g0644200	None		4.34
gene:Os06g0129900	Os06g0129900	LOC_Os06g03930.1	cytochrome P450 86A1, putative, expressed	4.31
C10728	Os01g0660200	LOC_Os01g47070.1	glycosyl hydrolase, putative, expressed	4.26
gene:Os03g0326200	Os03g0326200	LOC_Os03g20970.1	phospholipid-transporting ATPase 1, putative, expressed	4.15
OsMAS	Os04g0179200	LOC_Os04g10010.1	sex determination protein tasselseed-2, putative, expressed	4.11
gene:Os10g0535800	Os10g0535800	LOC_Os10g39100.1	uncharacterized Cys-rich domain containing protein, putative, expressed	4.10
gene:Os02g0102400	Os02g0102400	LOC_Os02g01230.1	ribosomal protein, putative, expressed	3.95
gene:Os07g0568000	Os07g0568000	LOC_Os07g38060.1	expressed protein	3.93
OsMSRA2.2	Os04g0482100	LOC_Os04g40620.1	peptide methionine sulfoxide reductase, putative, expressed	3.92
gene:Os07g0635150	Os07g0635150	None		3.86
gene:Os03g0351700	Os03g0351700	LOC_Os03g22820.1	expressed protein	3.81

Table S1. Continued

gene:Os08g0173600	Os08g0173600	LOC_Os08g07690.1	expressed protein	3.68
gene:Os01g0934300	Os01g0934300	LOC_Os01g70810.1	homeobox domain containing protein, expressed	3.68
gene:Os11g0514500	Os11g0514500	LOC_Os11g31540.1	BRASSINOSTEROID INSENSITIVE 1-associated receptor kinase 1 precursor, putative, expre	3.65
gene:Os05g0444200	Os05g0444200	LOC_Os05g37190.1	ZOS5-08 - C2H2 zinc finger protein, expressed	3.57
gene:Os04g0173800	Os04g0173800	LOC_Os04g09390.1	HEV3 - Hevein family protein precursor, expressed	3.50
OsSCP64	Os11g0643400	LOC_Os11g42390.1	OsSCP64 - Putative Serine Carboxypeptidase homologue, expressed	3.49
gene:Os02g0209400	Os02g0209400	LOC_Os02g11870.1	expressed protein	3.46
gene:Os01g0660000	Os01g0660000	None		3.46
gene:Os05g0546400	Os05g0546400	LOC_Os05g46840.1	proline-rich protein, putative, expressed	3.44
gene:Os01g0341750	Os01g0341750	None		3.43
gene:Os05g0112800	Os05g0112800	LOC_Os05g02200.1	cysteine-rich repeat secretory protein 55 precursor, putative, expressed	3.42
gene:Os05g0522600	Os05g0522600	LOC_Os05g44770.1	receptor-like protein kinase 5 precursor, putative, expressed	3.38
gene:Os07g0538000	Os07g0538000	LOC_Os07g35350.1	glucan endo-1,3-beta-glucosidase precursor, putative, expressed	3.37
gene:Os07g0125201	Os07g0125201	LOC_Os07g03319.1	SCP-like extracellular protein, expressed	3.27
OsPR1#072	Os07g0127500	LOC_Os07g03580.1	SCP-like extracellular protein, expressed	3.27
gene:Os07g0635200	Os07g0635200	LOC_Os07g44110.1	cytochrome P450 72A1, putative, expressed	3.26
gene:Os07g0126500	Os07g0126500	LOC_Os07g03499.1	SCP-like extracellular protein, expressed	3.24
OsPYL/RCAR2	Os02g0226801	LOC_Os02g13330.1	bet v I allergen family protein, putative, expressed	3.19
OsABCG48	Os11g0587600	LOC_Os11g37700.1	pleiotropic drug resistance protein, putative, expressed	3.15
gene:Os12g0629700	Os12g0629700	None		3.11
brd1	Os03g0602300	LOC_Os03g40540.1	cytochrome P450, putative, expressed	3.09
gene:Os02g0209300	Os02g0209300	LOC_Os02g11859.1	expressed protein	3.07
gene:Os05g0403300	Os05g0403300	LOC_Os05g33430.1	xyloglucanase inhibitor, putative, expressed	3.01
RASI	Os04g0526600	LOC_Os04g44470.1	KUN1 - Kunitz-type trypsin inhibitor precursor, expressed	3.01
gene:Os05g0130100	Os05g0130100	LOC_Os05g03920.1	TKL_IRAK_DUF26-lf.3 - DUF26 kinases have homology to DUF26 containing loci, expresse	3.01
OsABCG44	Os08g0384500	LOC_Os08g29570.1	pleiotropic drug resistance protein 3, putative, expressed	2.98
gene:Os04g0634000	Os04g0634000	LOC_Os04g54140.1	receptor-like kinase, putative, expressed	2.94
OsGELP11	Os01g0216900	LOC_Os01g11790.1	GDSL-like lipase/acylhydrolase, putative, expressed	2.94
OsGELP57	Os04g0561800	LOC_Os04g47390.1	GDSL-like lipase/acylhydrolase, putative, expressed	2.93
OsPR5_4	Os12g0628600	LOC_Os12g43380.1	thaumatin, putative, expressed	2.91
OsLEA3-1	Os05g0542500	LOC_Os05g46480.1	late embryogenesis abundant protein, group 3, putative, expressed	2.82
gene:Os02g0740600	Os02g0740600	LOC_Os02g50710.1	expressed protein	2.77
RF2b	Os03g0336200	LOC_Os03g21800.1	bZIP transcription factor family protein, putative, expressed	2.76
gene:Os05g0576600	Os05g0576600	LOC_Os05g50100.1	expressed protein	2.75
OsKOD1	Os04g0507950	None		2.75
prx92	Os06g0695300	LOC_Os06g48010.1	peroxidase precursor, putative, expressed	2.69
ADC2	Os04g0107600	LOC_Os04g01690.1	pyridoxal-dependent decarboxylase protein, putative, expressed	2.66
gene:Os07g0126100	Os07g0126100	LOC_Os07g03409.1	SCP-like extracellular protein, expressed	2.64
gene:Os06g0715500	Os06g0715500	LOC_Os06g50154.1	translocon-associated protein subunit alpha precursor, putative, expressed	2.64
OsRLCK255	Os08g0457400	LOC_Os08g35600.1	tyrosine protein kinase domain containing protein, putative, expressed	2.63
PDIL1_2	Os02g0554900	LOC_Os02g34940.1	OsPDIL1-3 protein disulfide isomerase PDIL1-3, expressed	2.61
OsNADPH1	Os04g0497000	LOC_Os04g41960.1	NADP-dependent oxidoreductase, putative, expressed	2.61
gene:Os02g0831300	Os02g0831300	LOC_Os02g58460.1	beta-catenin-like protein 1, putative, expressed	2.58
OsLOX10	Os11g0575600	LOC_Os11g36719.1	lipoxygenase, putative, expressed	2.52
OsASNase2	Os04g0650700	LOC_Os04g55710.1	transposon protein, putative, unclassified, expressed	2.51
OsPR4d	Os11g0591800	LOC_Os11g37940.1	WIP2 - Wound-induced protein precursor, expressed	2.51
gene:Os02g0205500	Os02g0205500	LOC_Os02g11070.1	3-ketoacyl-CoA synthase, putative, expressed	2.50
gene:Os11g0246166	Os11g0246166	None		2.48
HB1	Os03g0233900	LOC_Os03g13140.1	non-symbiotic hemoglobin 2, putative, expressed	2.47
OsPLDalpha4	Os06g0604200	LOC_Os06g40170.1	phospholipase D, putative, expressed	2.46
OsPGL32	Os01g0623600	LOC_Os01g43490.1	polygalacturonase, putative, expressed	2.44
gene:Os04g0326100	Os04g0326100	LOC_Os04g25970.1	cytokinin-O-glucosyltransferase 2, putative, expressed	2.42
gene:Os08g0158200	Os08g0158200	LOC_Os08g06170.1	berberine and berberine like domain containing protein, expressed	2.42
gene:Os09g0564400	Os09g0564400	LOC_Os09g39090.1	vignain precursor, putative, expressed	2.34
gene:Os06g0211600	Os06g0211600	LOC_Os06g10910.1	xyloglucan fucosyltransferase, putative, expressed	2.34
OsDjC29	Os03g0323600	LOC_Os03g20730.1	chaperone protein dnaJ, putative, expressed	2.34
OsPR5_2	Os03g0661600	LOC_Os03g45960.1	thaumatin, putative, expressed	2.34
OsCDAP1	Os07g0162400	LOC_Os07g06830.1	gibberellin receptor GID1L2, putative, expressed	2.32
NAC122	Os11g0126900	LOC_Os11g03300.1	NAC domain transcription factor, putative, expressed	2.30
gene:Os03g0772600	Os03g0772600	LOC_Os03g56160.1	lectin-like receptor kinase 7, putative, expressed	2.30
OsFKBP57	Os01g0562400	LOC_Os01g38180.1	peptidyl-prolyl isomerase, putative, expressed	2.27
OsKMD4	Os11g0246200	LOC_Os11g14140.1	OsFBK25 - F-box domain and kelch repeat containing protein, expressed	2.26
gene:Os03g0757000	Os03g0757000	LOC_Os03g55010.1	UDP-glucoronosyl and UDP-glucosyl transferase domain containing protein, expressed	2.26
gene:Os03g0388600	Os03g0388600	LOC_Os03g27090.1	MYB family transcription factor, putative, expressed	2.25
gene:Os10g0439100	Os10g0439100	LOC_Os10g30330.1	expansin precursor, putative, expressed	2.25
gene:Os08g0267300	Os08g0267300	LOC_Os08g16660.1	aspartic proteinase nepenthesin precursor, putative, expressed	2.24
gene:Os03g0726800	Os03g0726800	LOC_Os03g51670.1	serine esterase family protein, putative, expressed	2.23
DEFL12	Os04g0522100	LOC_Os04g44130.1	DEF12 - Defensin and Defensin-like DEFL family, expressed	2.22
OsGSTU41	Os01g0950000	LOC_Os01g72160.1	glutathione S-transferase, putative, expressed	2.22
OsRMC	Os04g0659300	LOC_Os04g56430.1	cysteine-rich receptor-like protein kinase, putative, expressed	2.20
gene:Os08g0111200	Os08g0111200	LOC_Os08g01940.1	non-lysosomal glucosylceramidase, putative, expressed	2.20

Table S1. Continued

gene:Os08g0206600	Os08g0206600	LOC_Os08g10570.1	bifunctional purine biosynthesis protein purH, putative, expressed	2.19
OsMT1g	Os12g0571000	LOC_Os12g38290.1	metallothionein, putative, expressed	2.19
Stt3a	Os04g0675500	LOC_Os04g57890.1	oligosaccharyl transferase, putative, expressed	2.18
gene:Os06g0634000	Os06g0634000	LOC_Os06g42754.1	expressed protein	2.18
HWH1	Os02g0621800	LOC_Os02g40840.1	alcohol oxidase, putative, expressed	2.18
gene:Os02g0206700	Os02g0206700	LOC_Os02g11640.1	UDP-glucoronosyl and UDP-glucosyl transferase, putative, expressed	2.15
gene:Os10g0189600	Os10g0189600	LOC_Os10g11200.1	aminotransferase, classes I and II, domain containing protein, expressed	2.15
gene:Os07g0563000	Os07g0563000	LOC_Os07g37580.1	diacylglycerol kinase, putative, expressed	2.14
CAL1	Os02g0629800	LOC_Os02g41904.1	DEF7 - Defensin and Defensin-like DEFL family, expressed	2.14
gene:Os05g0111200	Os05g0111200	LOC_Os05g02060.1	mitochondrial import inner membrane translocase subunit Tim17, putative, expressed	2.12
gene:Os06g0652600	Os06g0652600	LOC_Os06g44280.1	retrotransposon protein, putative, Ty3-gypsy subclass, expressed	2.12
gene:Os02g0705400	Os02g0705400	LOC_Os02g47650.1	universal stress protein domain containing protein, putative, expressed	2.12
OsEXPA11	Os01g0274500	LOC_Os01g16770.1	expansin precursor, putative, expressed	2.11
RBBI3-3	Os01g0124401	LOC_Os01g03360.1	BBTI5 - Bowman-Birk type bran trypsin inhibitor precursor, expressed	2.11
gene:Os06g0716100	Os06g0716100	LOC_Os06g50230.1	expressed protein	2.10
gene:Os01g0300200	Os01g0300200	LOC_Os01g19450.1	ATP-citrate synthase subunit 1, putative, expressed	2.09
NAC131	Os12g0123700	LOC_Os12g03040.1	no apical meristem protein, putative, expressed	2.09
gene:Os03g0434400	Os03g0434400	LOC_Os03g32040.1	phenazine biosynthesis protein, putative, expressed	2.09
GRF1	Os02g0776900	LOC_Os02g53690.1	growth regulating factor protein, putative, expressed	2.09
D11	Os04g0469800	LOC_Os04g39430.1	cytochrome P450, putative, expressed	2.09
OsLTP1.9	Os11g0115100	LOC_Os11g02350.1	LTPL25 - Protease inhibitor/seed storage/LTP family protein precursor, expressed	2.09
OsPTR3_1	Os10g0470700	LOC_Os10g33210.1	peptide transporter PTR3-A, putative, expressed	2.08
gene:Os05g0318600	Os05g0318600	LOC_Os05g25430.1	receptor-like protein kinase At3g46290 precursor, putative, expressed	2.08
AP2/EREBP129	Os01g0141000	LOC_Os01g04800.1	B3 DNA binding domain containing protein, expressed	2.08
gene:Os01g0947000	Os01g0947000	LOC_Os01g71860.1	glycosyl hydrolases family 17, putative, expressed	2.08
gene:Os03g0129800	Os03g0129800	LOC_Os03g03730.1	regulatory protein, putative, expressed	2.07
gene:Os11g0199801	Os11g0199801	LOC_Os11g09350.1	expressed protein	2.06
gene:Os10g0524700	Os10g0524700	LOC_Os10g38090.1	cytochrome P450, putative, expressed	2.06
gene:Os01g0693300	Os01g0693300	LOC_Os01g49820.1	lipid phosphatase protein, putative, expressed	2.06
RAC1	Os01g0229400	LOC_Os01g12900.1	ras-related protein, putative, expressed	2.04
OsUGT98B1	Os01g0176000	LOC_Os01g08090.1	flavonol-3-O-glycoside-7-O-glucosyltransferase 1, putative, expressed	2.03
Osr9-LOX1	Os03g0699700	LOC_Os03g49260.1	lipoxygenase, putative, expressed	2.03
OsTHI29	Os03g0247200	LOC_Os03g14300.1	THION29 - Plant thionin family protein precursor, expressed	2.01
gene:Os06g0290701	Os06g0290701	None		2.01
gene:Os06g0676700	Os06g0676700	LOC_Os06g46340.1	glycosyl hydrolase, family 31, putative, expressed	2.01

Table S2. Genes down-regulated in 10 μM TIS108-treated rice

Table S2 Down-regulated genes of 10 μM TIS108-treated rice				
Gene symbol	RAP ID	MSU ID	Description	Fold change
gene:Os05g0122600	Os05g0122600	LOC_Os05g03120.1	retrotransposon protein, putative, unclassified, expressed	-723.50
EIP11	Os08g0476300	LOC_Os08g37130.1	oxidoreductase, short chain dehydrogenase/reductase family domain containing protein, expressed	-473.03
gene:Os04g0488600	Os04g0488600	LOC_Os04g41150.1	DUF565 domain containing protein, putative, expressed	-459.00
gene:Os05g0393400	Os05g0393400	LOC_Os05g32680.1	PAC, putative, expressed	-194.64
gene:Os03g0335500	Os03g0335500	LOC_Os03g21730.1	receptor-like protein kinase precursor, putative, expressed	-147.11
gene:Os07g0178950	Os07g0178950	None		-57.10
gene:Os10g0487400	Os10g0487400	LOC_Os10g34590.1	zinc finger, C3HC4 type domain containing protein, expressed	-37.62
gene:Os04g0632400	Os04g0632400	None	#N/A	-26.10
prx7	Os07g0157000	LOC_Os07g06300.1	ethylene-insensitive protein 2, putative, expressed	-24.13
RR7	Os07g0449700	LOC_Os07g26720.1	OsRR7 type-A response regulator, expressed	-22.52
gene:Os07g0156467	Os07g0156467	LOC_Os07g06190.1	ethylene-insensitive protein 2, putative, expressed	-21.16
gene:Os07g0633100	Os07g0633100	LOC_Os07g43940.1	X8 domain containing protein, expressed	-18.72
OMT7	Os03g0708100	LOC_Os03g50040.1	phytanoyl-CoA dioxygenase, putative, expressed	-16.91
gene:Os11g0495950	Os11g0495950	LOC_Os11g30310.1	reticuline oxidase-like protein precursor, putative, expressed	-15.77
OsPKS16	Os10g0158400	LOC_Os10g07040.1	chalcone synthase, putative, expressed	-14.00
gene:Os07g0107583	Os07g0107583	None		-13.57
gene:Os05g0435300	Os05g0435300	LOC_Os05g35960.1	expressed protein	-13.55
gene:Os12g0173125	Os12g0173125	None		-12.03
gene:Os02g0593700	Os02g0593700	LOC_Os02g38050.1	joka2, putative, expressed	-11.51
CHS_2	Os05g0212900	LOC_Os05g12210.1	chalcone synthase, putative, expressed	-10.76
gene:Os12g0614201	Os12g0614201	None		-10.19
OsPKS07	Os05g0213100	LOC_Os05g12240.1	chalcone synthase, putative, expressed	-8.57
gene:Os01g0164075	Os01g0164075	LOC_Os01g07030.1	POEI40 - Pollen Ole e I allergen and extensin family protein precursor, expressed	-8.39
OsbHLH130	Os12g0589000	LOC_Os12g39850.1	helix-loop-helix DNA-binding domain containing protein, expressed	-7.82
OsPME9	Os02g0783000	LOC_Os02g54190.1	pectinesterase, putative, expressed	-7.50
PRX118	Os08g0302000	LOC_Os08g20730.1	peroxidase precursor, putative, expressed	-7.50
OSH1	Os03g0727000	LOC_Os03g51690.1	Homeobox domain containing protein, expressed	-6.45
gene:Os11g0156000	Os11g0156000	LOC_Os11g05740.1	B3 DNA binding domain containing protein, expressed	-6.33
OsCCR_1	Os08g0277200	LOC_Os08g17500.1	cinnamoyl-CoA reductase, putative, expressed	-6.10
OsalphaCA5	Os08g0424100	LOC_Os08g32840.1	bifunctional monodehydroascorbate reductase and carbonic anhydrasenectarin-3 precursor, putativ	-5.68
gene:Os11g0156000	Os11g0156000	LOC_Os11g05740.1	B3 DNA binding domain containing protein, expressed	-5.33
GLP8-4	Os08g0189300	LOC_Os08g08980.1	cupin domain containing protein, expressed	-5.28
gene:Os12g0614100	Os12g0614100	LOC_Os12g41970.1	lipase class 3 family protein, putative, expressed	-5.05
OsSub33	Os04g0120300	LOC_Os04g02980.1	OsSub33 - Putative Subtilisin homologue, expressed	-4.88
OsLLA8	Os10g0191100	LOC_Os10g11370.1	LTPL87 - Protease inhibitor/seed storage/LTP family protein precursor, putative, expressed	-4.65
gene:Os11g0289700	Os11g0289700	LOC_Os11g18570.1	cytochrome P450, putative, expressed	-4.63
gene:Os01g0163450	Os01g0163450	None		-4.28
GLP8-3	Os08g0189200	LOC_Os08g08970.1	Cupin domain containing protein, expressed	-3.90
OsCrRLK1L1	Os03g0759600	LOC_Os03g55210.1	TKL_IRAK_CrRLK1L-1.1 - The CrRLK1L-1 subfamily has homology to the CrRLK1L homolog, expressed	-3.88
gene:Os02g0124599	Os02g0124599	LOC_Os02g03210.1	FAD-binding and arabino-lactone oxidase domains containing protein, putative, expressed	-3.75
gene:Os10g0538450	Os10g0538450	None		-3.73
gene:Os06g0169001	Os06g0169001	LOC_Os06g07250.1	jacalin-like lectin domain containing protein, expressed	-3.69
gene:Os02g0184800	Os02g0184800	None		-3.50
OsTIP4_1	Os01g0232000	LOC_Os01g13120.1	aquaporin protein, putative, expressed	-3.44
OsERF#039	Os01g0200600	LOC_Os01g10370.1	AP2 domain containing protein, expressed	-3.35
gene:Os10g0450800	Os10g0450800	LOC_Os10g31320.1	retrotransposon protein, putative, unclassified, expressed	-3.30
OsIAA9	Os02g0805100	LOC_Os02g56120.1	OsIAA9 - Auxin-responsive Aux/IAA gene family member, expressed	-3.25
gene:Os09g0364800	Os09g0364800	LOC_Os09g20000.1	heavy metal-associated domain containing protein, expressed	-3.24
OsPP2C49	Os05g0457200	LOC_Os05g38290.1	protein phosphatase 2C, putative, expressed	-3.24
gene:Os05g0148800	Os05g0148800	LOC_Os05g05610.1	expressed protein	-3.24
gene:Os11g0303300	Os11g0303300	LOC_Os11g19780.1	O-methyltransferase ZRP4, putative	-3.18
gene:Os01g0191200	Os01g0191200	LOC_Os01g09540.1	HAD superfamily phosphatase, putative, expressed	-3.15
gene:Os03g0281466	Os03g0281466	LOC_Os03g17310.1	calcium-transporting ATPase, endoplasmic reticulum-type, putative, expressed	-3.10
gene:Os06g0179500	Os06g0179500	LOC_Os06g08120.1	plant protein of unknown function domain containing protein, expressed	-3.10
gene:Os01g0228450	Os01g0228450	None		-3.10
gene:Os05g0588900	Os05g0588900	LOC_Os05g51130.1	mitochondrial chaperone BCS1, putative, expressed	-3.05
gene:Os02g0596300	Os02g0596300	LOC_Os02g38290.1	cytochrome P450, putative, expressed	-2.98
gene:Os11g0635300	Os11g0635300	LOC_Os11g41680.1	cytochrome P450, putative, expressed	-2.93
OsSAUR33	Os08g0452500	LOC_Os08g35110.1	OsSAUR33 - Auxin-responsive SAUR gene family member, expressed	-2.91
gene:Os03g0740200	Os03g0740200	LOC_Os03g52940.1	expressed protein	-2.87
gene:Os04g0644100	Os04g0644100	LOC_Os04g55120.1	jp18, putative, expressed	-2.84
gene:Os09g0412700	Os09g0412700	LOC_Os09g24620.1	expressed protein	-2.81
gene:Os02g0542600	Os02g0542600	None		-2.74
OsTPP1	Os02g0661100	LOC_Os02g44230.1	CPuORF22 - conserved peptide uORF-containing transcript, expressed	-2.71
gene:Os03g0700450	Os03g0700450	None		-2.70
gene:Os06g0567433	Os06g0567433	None		-2.64
OsDXS	Os07g0190000	LOC_Os07g09190.1	transketolase, putative, expressed	-2.61
gene:Os04g0400600	Os04g0400600	LOC_Os04g32820.1	expressed protein	-2.61

Table S2. Continued

OsTPP1	Os02g0661100	LOC_Os02g44230.1	CPuORF22 - conserved peptide uORF-containing transcript, expressed	-2.60
CHS_1	Os04g0103900	LOC_Os04g01354.1	chalcone synthase, putative, expressed	-2.58
gene:Os12g0614200	Os12g0614200	LOC_Os12g41980.1	lipase class 3 family protein, putative, expressed	-2.54
OsOPR9	Os01g0370000	LOC_Os01g27240.1	12-oxophytodienoate reductase, putative, expressed	-2.53
gene:Os04g0538400	Os04g0538400	LOC_Os04g45520.1	integral membrane protein, putative, expressed	-2.49
gene:Os01g0588100	Os01g0588100	LOC_Os01g40560.1	hypersensitive-induced response protein, putative, expressed	-2.41
gene:Os01g0916100	Os01g0916100	LOC_Os01g68740.1	keratin, type I cytoskeletal 9, putative, expressed	-2.40
gene:Os01g0926400	Os01g0926400	LOC_Os01g70180.1	exostosin family domain containing protein, expressed	-2.39
OsPUB39	Os06g0248500	LOC_Os06g13870.1	U-box protein CMPG1, putative, expressed	-2.38
OsXTH13	Os02g0280200	LOC_Os02g17880.1	glycosyl hydrolases family 16, putative, expressed	-2.37
gene:Os09g0248900	Os09g0248900	LOC_Os09g07440.1	retrotransposon protein, putative, unclassified, expressed	-2.35
gene:Os03g0242300	Os03g0242300	LOC_Os03g13870.1	expressed protein	-2.34
gene:Os07g0638801	Os07g0638801	None		-2.33
ICS1	Os09g0361500	LOC_Os09g19734.1	isochorismate synthase 1, chloroplast precursor, putative, expressed	-2.28
gene:Os02g0628200	Os02g0628200	LOC_Os02g41780.1	transporter-related, putative, expressed	-2.23
gene:Os04g0630400	Os04g0630400	LOC_Os04g53810.1	leucoanthocyanidin reductase, putative, expressed	-2.23
Os-PHT2	Os06g0185300	LOC_Os06g08610.1	transferase family protein, putative, expressed	-2.19
gene:Os04g0578300	Os04g0578300	LOC_Os04g48870.1	nitrilase-associated protein, putative, expressed	-2.19
prx105	Os07g0638600	LOC_Os07g44460.1	peroxidase precursor, putative, expressed	-2.19
OsMC8	Os03g0389100	LOC_Os03g27190.1	ICE-like protease p20 domain containing protein, putative, expressed	-2.13
gene:Os04g0550866	Os04g0550866	None		-2.12
gene:Os01g0369950	Os01g0369950	None		-2.11
OsCDAP2	Os07g0162700	LOC_Os07g06860.1	gibberellin receptor GID1L2, putative, expressed	-2.11
OsPIP2_4	Os04g0521100	LOC_Os04g44060.1	aquaporin protein, putative, expressed	-2.10
gene:Os01g0700500	Os01g0700500	LOC_Os01g50490.1	cytochrome P450, putative, expressed	-2.09
gene:Os01g0164300	Os01g0164300	LOC_Os01g07060.1	POEI43 - Pollen Ole e I allergen and extensin family protein precursor, putative, expressed	-2.07
gene:Os03g0760500	Os03g0760500	LOC_Os03g55260.1	cytochrome P450, putative, expressed	-2.06
prx68	Os05g0135000	LOC_Os05g04450.1	peroxidase precursor, putative, expressed	-2.06
gene:Os03g0134500	Os03g0134500	LOC_Os03g04190.1	cytochrome P450, putative, expressed	-2.06
gene:Os06g0199700	Os06g0199700	LOC_Os06g09920.1	expressed protein	-2.05
gene:Os10g0162842	Os10g0162842	None		-2.05
gene:Os05g0355700	Os05g0355700	LOC_Os05g28770.1	GCRP9 - Glycine and cysteine rich family protein precursor, expressed	-2.04
gene:Os06g0133600	Os06g0133600	LOC_Os06g04250.1	phosphate-induced protein 1 conserved region domain containing protein, expressed	-2.04
gene:Os12g0135800	Os12g0135800	LOC_Os12g04150.1	alpha/beta hydrolase fold, putative, expressed	-2.04
OsIAA20	Os06g0166500	LOC_Os06g07040.1	OsIAA20 - Auxin-responsive Aux/IAA gene family member, expressed	-2.03
NaT	Os05g0382200	LOC_Os05g31730.1	transporter, monovalent cation:proton antiporter-2 family, putative, expressed	-2.02
gene:Os11g0644700	Os11g0644700	LOC_Os11g42500.1	dirigent, putative, expressed	-2.01
OsStr7	Os12g0428000	LOC_Os12g24020.1	rhodanese-like domain containing protein, putative, expressed	-2.01

Table S3. Expression of strigolactone- and tillering-related genes

Table S3 Expression of strigolactone- and tillering-related genes				
Genes	RAP ID	Variant	Fold change	FDR p-value
D27 9−cis/all−trans−β−carotene isomerase	Os11g0587000	D27_1	−1.82	0.69
		D27_2	−1.12	1.00
D10 Carotenoid cleavage dioxygenase8	Os01g0746400	D10_1	−1.68	0.07
D17 Carotenoid cleavage dioxygenase7	Os04g0550600	HED1_1	−1.75	0.05
MAX1 (Os5100) Cytochrome P450	Os06g0565100	Os5100_1	−1.31	0.61
MAX1 (Os1900) Cytochrome P450	Os02g0221900	Os1900_1	−66.14	0.45
		Os1900_2	−1.38	1.00
		Os1900_3	1.06	1.00
MAX1 (Os900) Cytochrome P450	Os01g0700900	SLB1_1	−1.07	1.00
		SLB1_2	−1.19	1.00
MAX1 (Os1400) Cytochrome P450	Os01g0701400	SLB2_1	−2.08	0.69
D3 F−box	Os06g0154200	D3_1	1.33	0.67
		D3_2	1.33	0.53
D14 α / β −Hydrolase	Os03g0203200	D14_1	1.39	1.00
		D14_2	−1.02	1.00
D53 Class I Clp ATPase	Os11g0104300	D53_1	−1.39	0.24
		D53_2	−1.10	1.00
FC1	Os03g0706500	FC1_1_1	−3.19	1.00
REP1	Os09g0410500	REP1_1	−1.03	1.00
OsIPA1	Os08g0509600	WFP_1	1.18	1.00
NSP1	Os03g0408600	NSP1_1	−1.21	0.83
NSP2	Os03g0263300	NSP2_1	−1.19	0.97

Table S4. List of primers used for RT-qPCR

Primer name	Primer sequence	Organism
UbiQ	F: GCCCAAGAAGAAGATCAAGAAC<br>R: AGATAACAACGGAAGCATAAAAGTC	Rice Nipponbare
OsMAX1-900	F: ATTGTCAGCGATCCACTTC<br>R: GCGCCGTTCTTGAAATTG	Rice Nipponbare
OsMAX1-1400	F: GGCAGGTGCTCAAGAGGATT<br>R: TTTTGTCCATCTGTCCCCCG	Rice Nipponbare
OsMAX1-1900	F: GTTCCCCATAGGCCACCTTC<br>R: GCATTGGCCACAATCACCAG	Rice Nipponbare
OsMAX1-5100	F: GTGATAAAGGAGGCGATGAG<br>R: CTTTGGGAGTGTGTAGCC	Rice Nipponbare
OsRubQ1	F: GGGTTCACAAGTCTGCCTATTTG<br>R: ACGGGACACGACCAAGGA	AMF – *R. irregularis*
OsPT11	F: GAGAAGTTCCCTGCTTCAAGCA<br>R: CATATCCCAGATGAGCGTATCATG	AMF – *R. irregularis*

Table S5. List of primers used for off-target study in rice Nipponbare

Off-target name	Primer sequence	Ta (°C) / expected fragment size (bp)
OS01G0279100	F: CTTCTTCAAGCCCAAGTTCATCT<br>R: CCAGACCAATCCCTTCATAGAATG	63.9 / 419
Intergenic region chr. 3: +19072231	F: AAAGCATGTCAAATGGGAGAAGTT<br>R: GATCTTCTCTTCACCCGACTTGTC	64.4 / 385
Intergenic region chr. 9: +20398520	F: ATCACACGCACCATCAGTCAATTA<br>R: GTAAACGGTATGTGAAAAGATGCC	63.3 / 363
Intergenic region chr. 9: +7106441	F: GATGGATTTGGCAAGCTCGA<br>R: TTAGGGGAGGCCATGATGAC	63.8 / 396

Data S1. RPKM values of all genes in RNA-seq
